# Metabolomic Assessment of Acute Cholestatic Injuries Induced by Thioacetamide and by Bile Duct Ligation, and the Protective Effects of Huang-Lian-Jie-Du-Decoction

**DOI:** 10.3389/fphar.2018.00458

**Published:** 2018-05-08

**Authors:** Dan-Dan Wei, Jun-Song Wang, Jin-Ao Duan, Ling-Yi Kong

**Affiliations:** ^1^Jiangsu Collaborative Innovation Center of Chinese Medicinal Resources Industrialization, National and Local Collaborative Engineering Center of Chinese Medicinal Resources Industrialization and Formulae Innovative Medicine, Nanjing University of Chinese Medicine, Nanjing, China; ^2^State Key Laboratory of Natural Medicines, Department of Natural Medicinal Chemistry, China Pharmaceutical University, Nanjing, China; ^3^Center for Molecular Metabolism, Nanjing University of Science and Technology, Nanjing, China

**Keywords:** metabolomics, Huang-Lian-Jie-Du-Decoction, acute cholestatic injuries, thioacetamide, bile duct ligation, orthogonal partial least squares-discriminant analysis

## Abstract

Huang-Lian-Jie-Du-Decoction, a traditional Chinese formula, has been reported to protect liver from various injuries. Two cholestasis models of rats induced by thioacetamide and by bile duct ligation were established and treated with Huang-Lian-Jie-Du-Decoction. Nuclear Magnetic Resonance-based urinary metabolic profiles were analyzed by orthogonal partial least squares discriminant analysis and univariate analysis to excavate differential metabolites associated with the injuries of the two models and the treatment effects of Huang-Lian-Jie-Du-Decoction. The two cholestatic models shared common metabolic features of excessive fatty acid oxidation, insufficient glutathione regeneration and disturbed gut flora, with specific characteristics of inhibited urea cycle and DNA damage in thioacetamide-intoxicated model, and perturbed Kreb's cycle and inhibited branched chain amino acid oxidation in bile duct ligation model. With good treatment effects, Huang-Lian-Jie-Du-Decoction could regain the balance of the disturbed metabolic status common in the two cholestasis injuries, e.g., unbalanced redox system and disturbed gut flora; and perturbed urea cycle in thioacetamide-intoxicated model and energy crisis (disturbed Kreb's cycle and oxidation of branched chain amino acid) in bile duct ligation model, respectively.

## Introduction

Traditional Chinese medicine (TCM) has been used for healthcare for thousands of years in China (Cheung, 2011). As different from western medicine in both methodology and philosophy, TCM concentrates on the overall functional state of the patient and the adjustment of its balance, and has gained ever-increasing interest worldwide, especially for the treatment of complex diseases (Jiang, 2005). The major challenge to the adoption and popularity of TCM is the mechanistic study of its efficacy due to its complicated principles (Wang R. et al., 2012). Similar to TCM, systems biology also treats the body as a whole and investigates the life holistically (Van der Greef, 2011), which was deemed as a technique that has the potential to make a real breakthrough in TCM (Qiu, 2007). Metabolomics, an important component of systems biology, is a sensitive tool to detect various stimuli by snapshotting metabolic status of the body (Nicholson et al., 1999; Lindon et al., 2003; Lucio, 2009). Metabolomics has been widely used for the assessment of the toxicity of TCM (Zira et al., 2013), disease diagnosis (Manna et al., 2013; Zeng et al., 2014), and phenotyping (An et al., 2012). The most common analytical techniques for metabolic profiling are nuclear magnetic resonance (NMR) spectroscopy, gas chromatography-mass spectrometry (GC-MS) and liquid chromatography mass spectrometry (LC-MS). Despite of a lower sensitivity than MS, NMR has been widely applied in metabolic profiling for its associated advantages such as non-destructive, non-biased, separation free, short analysis time, and rich in structural information (Barba et al., 2008; Wishart, 2008; Wheelock et al., 2013).

Liver is critical for bile formation, amino acid utilization and ammonia detoxification, and is also the tissue where glycolysis, gluconeogenesis, and the synthesis of certain plasma proteins happen (Beyoǧlu and Idle, 2013). The metabolic rate and energy production and consumption in liver are extremely fast, which makes liver vulnerable to drugs and toxins and sensitive to many pathological insults (Whitfield et al., 2005; Vinaixa et al., 2010). Flowing from liver to duodenum through hepatobiliary transport systems, bile plays an important role in the digestion of fats. Any obstruction in bile duct systems could result in an accumulation of bile acids and other harmful compounds, leading to cholestatic injury. Cholestatic injuries could be formed by various means. The hepatoxin thioacetamide could inhibit bile secretion, producing intrahepatic cholestatic injuries (Shukla et al., 1992); ligation of the common bile duct could lead to a mechanical blockage in the duct system, inducing obstructive extrahepatic cholestatic injuries.

Huang-Lian-Jie-Du-Decoction (HLJDD; also known as oren-gedoku-to or Hwangryun-Hae-Dok-Decotion in Japan), representative ancient antipyretic and detoxifying traditional Chinese medicine recipe, is comprised of *Rhizoma coptidis, Radix scutellariae, Cortex phellodendri*, and *Fructus gardeniae* in a weight ratio of 3:2:2:3, functioning as clearing “heat” and removing “poison.” It has been used to treat in clinical for nearly two thousand years, was mentioned firstly in Wang Tao's “Wai Tai Mi Yao” two thousand years ago, it has been widely used to treat inflammatory-related diseases, such as liver injury (Wei et al., 2015; Lv et al., 2017), ischemic stroke (Wang et al., 2014; Zhang et al., 2017a), arthritis (Hu et al., 2013), and dermatitis (Chen et al., 2016). Its component herb, *Rhizoma coptidis* and its main alkaloid berberine exhibited hepatoprotective activity on acute hepatotoxicity and liver fibrosis induced by various drugs and chemicals, e.g., acetaminophen (Janbaz and Gilani, 2000), carbon tetrachloride (Ye et al., 2009; Feng et al., 2010), diethylnitrosamine and phenobarbital (Zhao et al., 2008). Their protection on liver injuries was mainly mediated by inhibition of various inflammatory factors: tumor necrosis factor-α, cyclooxygenase-2, and inducible nitric oxide synthase expression (Zhao et al., 2008; Domitrovi et al., 2011). Baicalin, a major flavone from RS, has also showed protective effect on liver injuries induced by acetaminophen (Jang et al., 2003), lipopolysaccharide/d-galactosamine (Liu et al., 2008; Wan et al., 2008), ischemia/reperfusion (Kim et al., 2010), and iron overloading (Zhao et al., 2005).

Cholestatic injury was associated with inflammation, and our previous study has demonstrated anti-inflammatory effects of HLJDD both *in vitro* and *in vivo* (Lu et al., 2011). HLJDD, its main herb and representative components have been indicated to possess therapeutic potential in the treatment of stroke (Wang et al., 2013, 2014; Zhang et al., 2015, 2016, 2017a,b; Zhu et al., 2018), chronic cholestasis injury (Wei et al., 2015) and multiple organ failure during sepsis (Liao et al., 2016a; Li et al., 2017; Lv et al., 2017; Xu et al., 2017). Herein the aim of the present work was to comparatively study the two cholestatic injuries induced by thioacetamide and by bile duct ligation, and their treatment by HLJDD using an NMR-based metabolomic approach. Multivariate analysis technique orthogonal partial least squares-discriminant analysis (OPLS-DA), combined with univariate analysis, was applied to excavate metabolic features associated with metabolic and mechanical cholestatic injuries of the two models and the treatment effects of HLJDD on them.

## Materials and methods

### Chemicals and reagents

*Rhizoma Coptidis, Radix Scutellariae, Cortex Phellodendri Chinensis*, and *Fructus Gardeniae* were obtained from Jiangsu medicine company (Nanjing, China) and were authenticated by Professor Jin-ao Duan, department of Resources and Authentication of Chinese Medicine, school of Pharmacy, Nanjing University of Chinese Medicine (Nanjing, China) as *Coptis teeta* Wall (Ranunculaceae), *Scutellaria baicalensis* Georgi (Lamiaceae), *Phellodendron chinense* Schneid. (Rutaceae) and *Gardenia jasminoides* Ellis (Rubiaceae), respectively. Voucher specimens were deposited at the herbarium of Jiangsu Collaborative Innovation Center of Chinese Medicinal Resources Industrialization, Nanjing University of Chinese Medicine, with the voucher number of RC201335, RS201323, CP201329, and FG201319.

Geniposidic acid, geniposide, chlorogenic acid, phellodendrine, magnoflorine, tetrahydropalmatine, coptisine, jatrorrhizine, baicalein, palmatine, berberine, baicalin and wogonin were bought from Tianjin Chemical Industrial Co. (Tianjin, China). Formic acid, methanol and acetonitrile were of HPLC grade, and were obtained from Merck KGaA (Darmstadt, Germany). Sodium 3-trimethylsilyl-1-(2,2,3,3-2H4) propionate was bought from Sigma Chemical Co. (St. Louis, MO, USA). Deuterium oxide (99.9%) was purchased from Sea Sky Bio Technology Co. Ltd (Beijing, China). Thioacetamide were bought from Shanghai Lingfeng Chemical Reagent Co., Ltd (Shanghai, China). All reagents were of analytical grade with purity over 98%. Ultra-pure distilled water was prepared from a Milli-Q purification system.

### Preparation of HLJDD extract solution and the reference solution

*Rhizoma Coptidis, Radix Scutellariae, Cortex Phellodendri Chinensis*, and *Fructus Gardeniae* in a weight ratio of 3:2:2:3, reaching a total weight of 5.0 kg, was dried, powdered, and extracted with 70% ethanol (1:10, 1:8 and 1:5, w/v) under reflux three times for 1 h each as our previous report (Wang et al., 2013). The extract solution was combined, filtered through 5-layer gauze to give a filtrate, which was concentrated on a rotary vacuum evaporator, and freeze-dried in a vacuum to collect HLJDD extract (1,525.432 g, yield: 30.51%). 1.00 mg HLJDD extract was accurately weighed and dissolved in 5.00 ml methanol to obtain a sample solution at the concentration of 200 μg/ml, while references of approximately 2 mg were accurately weighed and placed in a 10 ml volumetric flask to prepare a mixture of stock solution, with concentrations of 209 μg/ml (geniposidic acid), 308 μg/ml (chlorogenic acid), 106 μg/ml (phellodendrine), 135 μg/ml (magnoflorine), 263 μg/ml (tetrahydropalmatine), 143 μg/ml (coptisine), 120 μg/ml (jatrorrhizine), 268 μg/ml (baicalein), 134 μg/ml (palmatine), 106 μg/ml (berberine), 83 μg/ml (baicalin), and 243 μg/ml (wogonin).

### Qualitative and quantitative analysis of HLJDD extract solution by ultra-high pressure liquid chromatography with a linear ion trap-high resolution orbitrap mass spectrometry system (UPLC-LTQ-orbitrap MS)

The UPLC analyses were performed on a Dionex Ultimate 3000 UPLC system (Thermo Fisher, Hopkinson, MA, USA), hyphenated with a linear ion trap orbitrap XL mass spectrometry system, which was equipped with an ESI source. Chromatographic separations were carried out using a Waters Acquity UPLC column (BEH C18 100 × 2.1 mm, 1.7 μm) at 30°C. The mobile phase was composition of 0.1% formic acid-water (v/v, phase A) and acetonitrile (phase B), in a gradient program: 0–1 min, 3% B; 1–10 min, 3–20% B; 10–25 min, 20–50% B; 25–27 min, 50–95% B. The flow rate was set at 0.4 ml/min and the injection volume was 2 μL.

The Orbitrap instrument was calibrated using a solution containing caffeine, methionine-arginine-phenylalanine-alanine, and Ultramark according to the manufacturer's instructions before use, and signals from each of the orbitrap outer electrodes were amplified by a differential amplifier and transformed into a frequency spectrum by fast Fourier transformation. Mass spectrometer was operated with an ESI probe in positive and negative ion modes, the mass range was between 100 and 1,000, and the resolution was 30,000. All data were acquired in centroid mode, and operation of the entire LC/MS instrumentation was controlled using LCQ Xcalibur software (Thermo Fisher Scientific, Waltham, MA, USA). Parameters of the ion source were as follows: capillary temperature and voltage at 275°C and 40 V, respectively, source voltage at 5 kV and tube lens voltage at 80 V. Nitrogen was used as the sheath gas with a flow rate of 8 arbitrary units.

### Animal treatment

Seventy-two male Sprague-Dawley rats (with an average age of 8 weeks and weight of 220–240 g), were bought from Laboratory Animal Research Center, Nanjing University (Nanjing, China). Rats were housed in a well-ventilated room at constant ambient temperature (25 ± 2°C) and air humidity (50 ± 10%) with a light/dark cycle of 12 h, and were allowed *ad libitum* access to water throughout the study. Animal experiments were reviewed, approved and conducted in accordance with guidelines set by National Institutes of Health and the Institutional Animal Care and Use Committee at Nanjing University of Chinese Medicine (approbation number: NJUCM2013068). Standard biosecurity procedures were followed throughout the study in a Good Laboratory Practice-compliant experimental trials platform in Nanjing University of Chinese medicine, which maintained a strict rodent control policy. All the rooms and housing environment (ie walls, floor, bars, bottle and roof of the cage) were completely disinfected every 3 days. All food, water and bedding were packed in a bulk sterilizing chamber and vacuum sterilized with a vacuum autoclave before use. All the operation of animal tissues, organs, body parts, carcasses waste, fluid, discarding urine, sharps and disposal of experimental refuse were in accordance with the Centers for Disease Control's recommendations and the Environmental Protection Agency's recommendations to prevent introduction of pathogens.

After acclimatization for a week, rats were randomly divided into six groups (*n* = 12): two model groups TAA (intoxicated with thioacetamide) and BDL (bile duct ligation), two HLJDD treatment groups THC (on TAA model) and BHD (on BDL model), and two control groups NC and HLD. The HLD, THC and BHD groups were intragastrically (i.g.) administrated with HLJDD extract suspended in 0.5% carboxymethyl cellulose sodium salt (1.47 g/kg/day), while the NC, TAA and BDL groups were administrated with an equivalent amount of vehicle for 1 week.

After an overnight fasting, the TAA and THC rats were injected intraperitoneally with thioacetamide (dissolved in saline, 200 mg/kg), the HLD and NC rats were intraperitoneally administrated with the same amount of saline. BDL and BHD rats were anesthetized by intraperitoneal injection with chloral hydrate (300 mg/kg) and kept in anesthetization throughout the experiment. After a midline incision in the stomach under sterile conditions, gastroduodenal ligament was exposed to allow the isolation of the common bile duct, which was ligated singly with 4-0 nylon silk sutures, followed by careful suturing of the peritoneum and muscle layers as well as the skin wound.

### Urine samples collection

All rats were *ad libitum* accessed to standard rat pellet diet and tap water. HLD, THC, and BHD rats were i.g. administrated with HLJDD solution once a day after the operation, while NC, TAA, and BDL rats were treated with an equivalent amount of vehicle. Animals were housed individually in metabolic cages to collect urine samples at the following time periods: 0–8 h (T1), 8–24 h (T2), 24–32 h (T3), 32–48 h (T4), 48–72 h (T5), 72–96 h (T6), 96–120 h (T7) and 120–144 h (T8) after treatment. All urine samples were collected over dry ice with the addition of sodium azide (0.1% w/v urine solution) to the collection vessel to minimize bacterial contamination. At the end of the collection, urine was centrifuged at 12,000 g, 10 min, at 4°C to remove food. All the urine samples for NMR spectroscopic analysis were stored in multiple aliquots at −80°C before use.

### Pathological assessment

Overnight fasted rats were euthanized at 48 and 144 h after operation, and hearts, livers, spleens, lungs, kidneys and brains tissues were collected, and then preserved in 10% buffered formalin. A series of adjacent 3-μm-thick sections were cut and stained with hematoxylin & eosin for independent histological assessment by an experienced pathologist from Jiangsu Provincial Institute of Materia Medica (Nanjing, China).

### Acquisition of 1H NMR spectra of urine

The deep-frozen urine samples were thawed at 4°C overnight, and then were vortexed to dissolve any precipitates. An aliquot of 400 μL urine was mixed with 200 μL phosphate buffered saline (0.2 M Na_2_HPO4 and 0.2 M NaH_2_PO4, pH 7.0), allowing to equilibrate for 10 min prior to centrifugation (12,000 g for 10 min, at 4°C). The supernatant was added with 50 μL Sodium 3-trimethylsilyl-1-(2,2,3,3-2H4) propionate solution (dissolved in deuterium oxide, 1 mg/ml).

All NMR spectra were recorded using a Bruker AV 500 MHz spectrometer, with a nuclear overhauser enhancement spectroscopy presaturation pulse sequence (relaxation delay-90°-t1-90°-tm-90°-acquire-FID). Typically, 64 free induction decay was collected into 32 K data points, using a spectral width of 10 kHz, an acquisition time per scan of 2.54 s, recycle delay of 2 s and a mixing time of 100 ms. With TopSpin software (version 3.0, Bruker Biospin, Germany), all the spectra were automatically phased and baseline corrected, and calibrated to Sodium 3-trimethylsilyl-1-(2,2,3,3-2H4) propionate at 0.00 ppm.

### Processing of NMR data

With the removal of signals of water and urea (4.2–5.9 ppm), the NMR spectra were aligned in R, a freely available, open-source software (R Development Core Team, http://cran.r-project.org/). Regions between 0.5 and 9.8 ppm were binned, probabilistic quotient normalized, mean-centered and pareto scaled in R prior to multivariate statistical analysis.

### Identification of metabolites

Aided by Statistical total correlation spectroscopy, metabolites were identified by comparison with the standard references in the Human Metabolome Database (www.hmdb.ca) under the help of Chenomx NMR Suite, version 4.0 (Chenomx Inc., Edmonton, Canada). These assignments were finally confirmed by 2D NMR techniques, e.g., ^1^H-^1^H total correlation spectroscopy and ^1^H-^13^C heteronuclear single-quantum correlation.

### Multivariate analysis

Supervised orthogonal partial least squares-discriminant analysis (OPLS-DA) was performed using scripts written in R. A repeated 2-fold cross-validation and permutation test (*n* = 2,000) were then performed to validate the OPLS-DA models. Goodness-of-fit (*R*^2^) and goodness of prediction (Q^2^) of established models was calculated: typically a model with a Q^2^ value of greater than 0.40 is considered good, and with Q^2^ values over 0.70 is robust (Lourenço et al., 2011). Receiver operating characteristic (ROC) plots were delineated to evaluate classification performance using R-package ROCR (http://rocr.bioinf.mpi-sb.mpg.de). The area under the ROC (AUROC) is a measure of the overall accuracy, with a value of 1.000 and 0.500 representing perfect discrimination and random classification, respectively.

### Univariate analysis

Levels of metabolites of each group throughout the experiment were calculated corresponding to the normalized average integration of area (Zira et al., 2013). To deduct the metabolic alterations of healthy rats among different time points, the variations of metabolites in HLD, TAA, BDL, THC, and BHD groups were normalized by NC group. Considering the potential effect of HLJDD on metabolic status of healthy rats, the variations of metabolites in THC and BHD groups were normalized by HLD group. According to the conformity to normality, the statistical significance was calculated using either parametric (*t*-test) or non-parametric statistical test (Wilcoxon signed rank test) for metabolites between groups using R, with *p* < 0.05 as significant.

## Results

### Histopathological inspection

**Figure 1:**
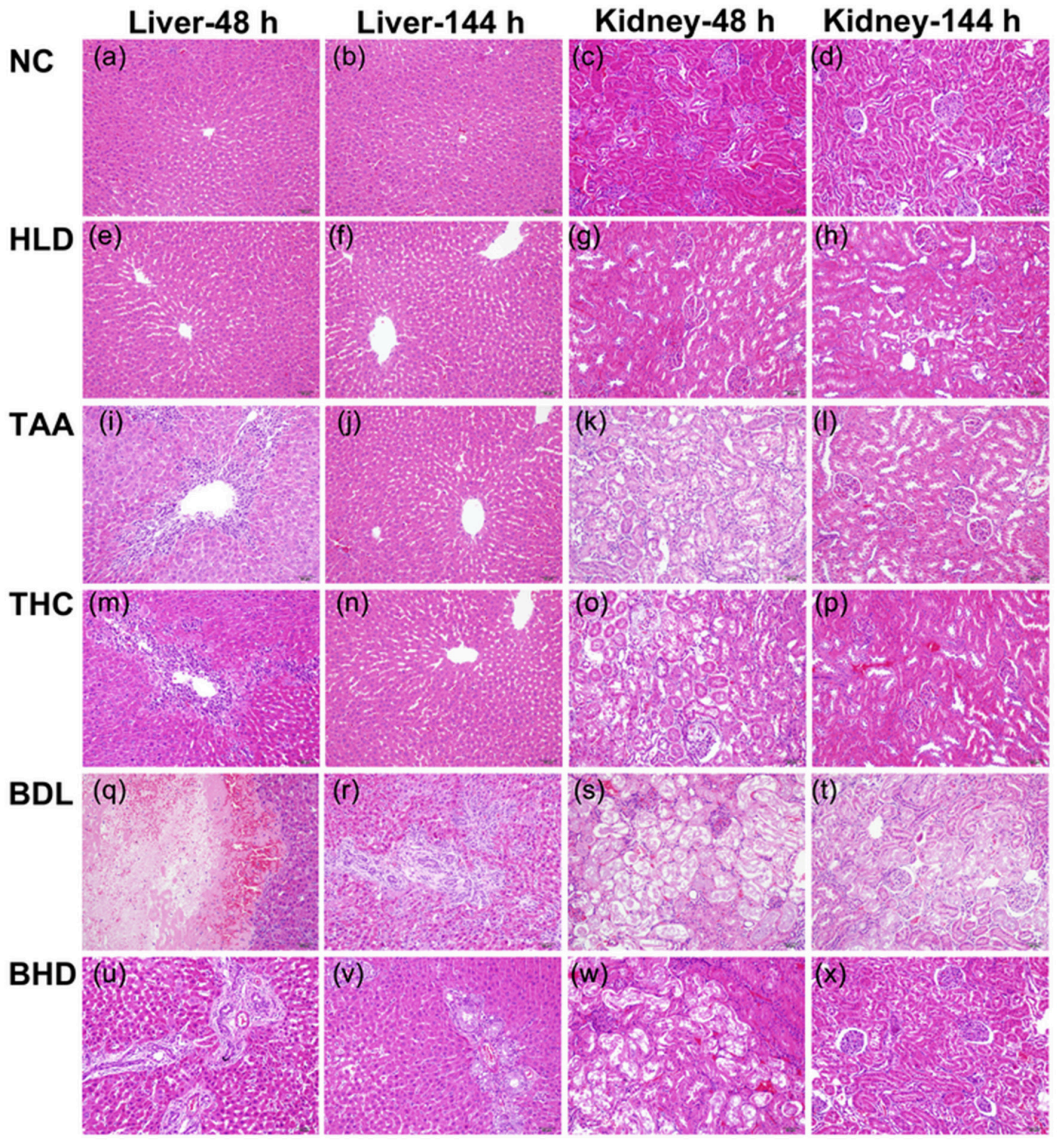
Treatment effect of Huang-lian-jie-du decoction (HLJDD) on the histology of livers and kidneys of thioacetamide-intoxicated (TAA) and bile duct ligation (BDL) rats sacrificed at 48 and 144 h. **(a–d)** Normal control rats (NC). **(e–h)** NC rats administrated with HLJDD (HLD): showing no signs of pathological change. **(i–l)** TAA rats: inflammatory foci in liver **(i)** and slightly swollen kidneys **(k)**. **(m–p)** TAA rats treated with HLJDD (THC): slight inflammatory cell infiltration in hepatocytes **(m,n)** and partial necrosis in kidneys **(o,p)**. **(q–t)** BDL rats: intense focal necrosis **(q)**, proliferation of small bile duct and fibrogensis **(r)**, collagenic exudation **(s)** and necrosis of epithelial cell **(t)**. **(u–x)** BDL rats treated with HLJDD (BHD): slight necrosis **(u)**, partial proliferation of small bile duct and fibrogensis **(v)**, mild collagenic exudation **(w)**, and no signs of pathological changes **(x)**.

It revealed no pathological alterations in hearts, spleens, lungs and brains for all groups and also no noticeable changes in the livers and kidneys (Figures 1a–h) of NC and HLD rats. At 48 h, the livers of TAA rats exhibited inflammatory foci, characteristics of monocytes in mesenchymal (Figure 1i), and kidneys of TAA rats were slightly swollen and lobulated with active glomerulogenesis (Figure 1k). The pathological alterations of livers and kidneys in TAA rats were both recovered at 144 h (Figures 1j,l). On the treatment of HLJDD, THC rats showed attenuated inflammatory cell infiltration in hepatocytes at 48 h (Figures 1m,n), in consistent with the previously reported anti-inflammatory effect of HLJDD (Lu et al., 2011). Slight necrosis was observed in kidneys of THC rats at 48 h (Figures 1o,p).

Livers of BDL rats exhibited severe hepatocellular injury with intense focal necrosis and degeneration at 48 h (Figure 1q), and striking cholangiocyte proliferation, stellate cell activation and fibrogensis at 144 h after operation (Figure 1r), in consistent with those previously reported (Chen et al., 2012). Renal slices of BDL rats presented significant intense collagenic exudation, around by severe necrosis of epithelial cell at 48 h (Figure 1s), and decreased volume of glomeruli at 144 h (Figure 1t). On the treatment of HLJDD, the collagenic exudation was strikingly attenuated in livers of BHD rats at 48 h (Figure 1u) and recovered at 144 h (Figure 1v), and renal slices exhibited slight necrosis and moderate cholangiocyte proliferation at 48 h (Figure 1w), which was recovered to normal at 144 h (Figure 1x). The pathological changes induced by hioacetamide and bile duct ligation were mainly in livers and kidneys. Thioacetamide produced a mild injury as compared with a relatively severer damage induced by bile duct ligation, both of which peaked at 48 h and attenuated at 144 h, suggesting a self-regulating and self-regenerating capacity of liver and kidney. HLJDD could ameliorate the lesions produced in both models.

### UPLC-LTQ-orbitrap MS analysis of HLJDD extract solution

**Figure 2:**
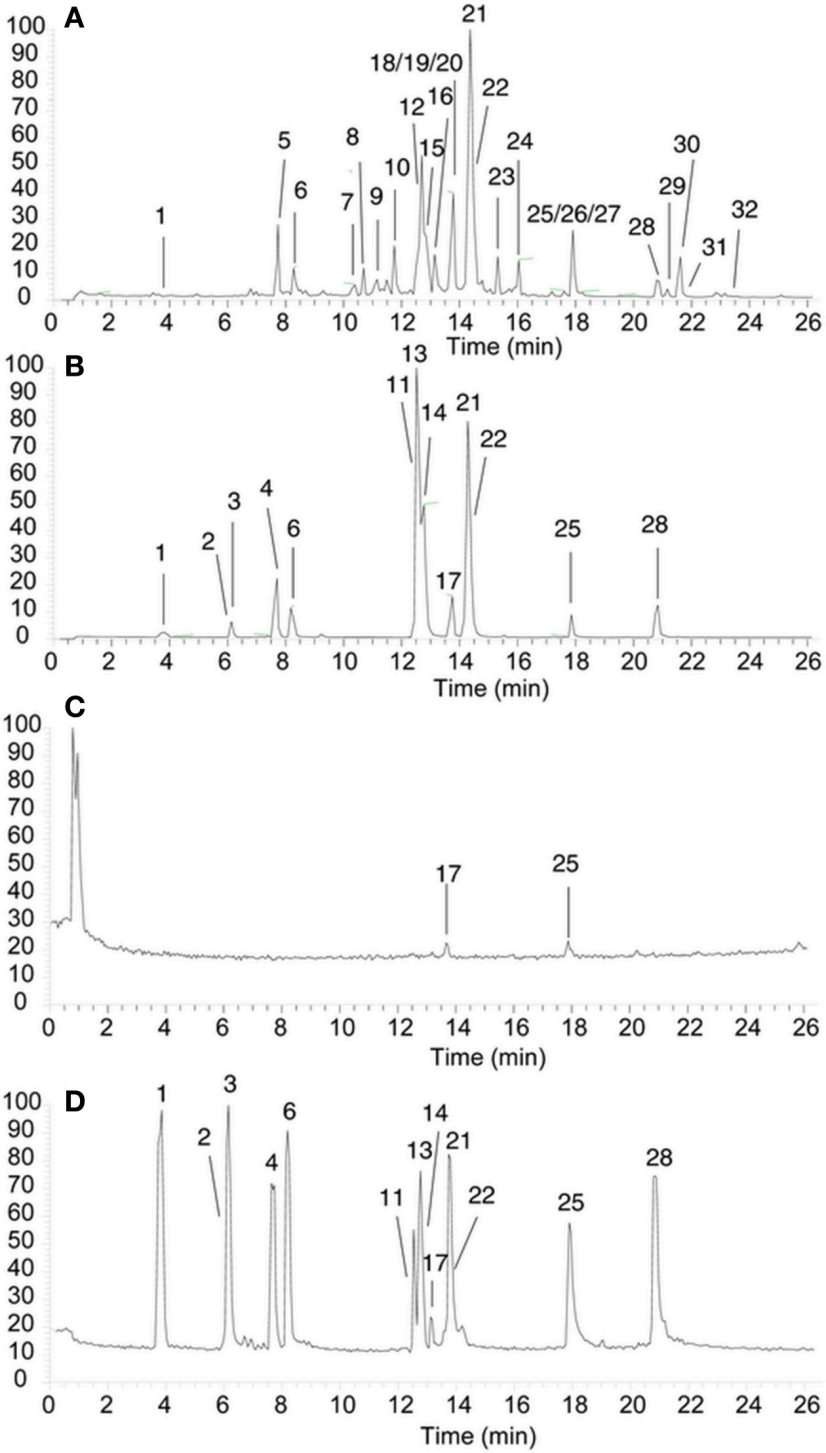
**(A,C)** UPLC chromatogram of the HLJDD extract with its secondary metabolites assigned. **(B,D)** The typical UPLC chromatograms of a mixture of thriteen standards (**A,B** in ESI+ mode, **C,D** in ESI- mode): Geniposidic acid (1, 3.82 min), geniposide (2, 6.13 min), chlorogenic acid (3, 6.25 min), phellodendrine (4, 7.71 min), magnoflorine (6, 8.30 min), tetrahydropalmatine (11, 12.65 min), coptisine (13, 12.67 min), jatrorrhizine (14, 12.83 min), baicalein (17, 13.79 min), palmatine (21, 14.28 min), berberine (22, 14.36 min), baicalin (25, 17.91 min), and wogonin (28, 20.67 min).

Thirty-two main base peaks in the chromatograms of HLJDD extract solution were successfully identified according to the retention time, accurate mono-molecular weight and by comparison with the standard references, including 2 phthalate of bis(2-ethylhexyl) phthalate and dioctyl phthalate, 2 phenols of chlorogenic acid and sinapyl aldehyde, 3 iridoid glycosides of geniposide, geniposidic acid and obacunone, 11 flavonoids of baicalin, baicalein-7-O-β-D-glucopyranoside, apigenin 7-O-β-D-glucuronide, baicalein, norwogonin, luteolin/kaempferol, wogonin, 3′,4′,5′-O-trimethyltricetin, prunetin, puararin, dihydrowogonin, and 14 alkaloids of phellodendrine, magnoflorine, thalifendine, berberastine, 8-oxypalmatine, tetrahydropalmatine, epiberberine, coptisine, jatrorrhizine, columbamine, berberrubine, worenine, palmatine and berberine (Figures 2A,C, Table 1). Thirteen standard reference chemcials were employed as external standard solutions, and calibration curves were generated for each reference by linear regression analysis to determine the contents in HLJDD extract (Table 2). Thirteen major components were successfully identified and determined: Geniposidic acid (1), geniposide (2), chlorogenic acid (3), phellodendrine (4), magnoflorine (6), tetrahydropalmatine (11), coptisine (13), jatrorrhizine (14), baicalein (17), palmatine (21), berberine (22), baicalin (25), and wogonin (28) (Figures 2B,D).

**Table 1:** Metabolites putatively identified by UPLC-LTQ-orbitrap-MS analysis in the extract of HLJDD.

**Peak NO**.	**Name**	**RT (min)**	**CompMW**	**m/z**		**DeltaPPM**	
				**ESI+**	**ESI−**	**ESI+**	**ESI−**
1	Geniposidic acid	3.82	374.1213	392.1533	373.1267	3	5
2	Chlorogenic acid	6.13	354.0951	355.1023	353.0908	1	3
3	Geniposide	6.25	388.1357	389.1429	/	3	/
4	Phellodendrine	7.71	342.1700	342.1723	340.1608	1	4
5	Sinapyl aldehyde	7.78	208.0729	209.0802	/	3	/
6	Magnoflorine	8.30	342.1700	342.1680	340.1608	5	4
7	Dioctyl phthalate	10.68	390.2759	391.2831	/	3	/
8	Thalifendine	11.09	322.1059	322.1059	/	0	/
9	Berberastine	11.42	351.1098	352.1171	/	1	/
10	8-Oxypalmatine	11.75	367.1413	368.1486	/	5	/
11	Tetrahydropalmatine	12.65	355.1762	356.1835		6	
12	Epiberberine	12.66	336.1230	336.1211	/	6	/
13	Coptisine	12.67	320.0917	320.0917	/	2	/
14	Jatrorrhizine	12.83	338.1387	338.1367	338.1439	6	1
15	Columbamine	12.83	337.1294	338.1367	/	1	/
16	Berberrubine	13.67	321.1000	322.1072	/	1	/
17	Baicalin	13.79	446.0838	447.0911	445.0804	2	5
18	Baicalein-7-b-D-glucopyranoside	13.83	432.1049	433.1122	431.0968	2	1
19	Puararin	13.83	416.1095	417.1168	/	3	/
20	Worenine	14.09	333.0992	334.1065	/	3	/
21	Palmatine	14.28	352.1543	352.1516	/	8	/
22	Berberine	14.36	336.1230	336.1210	/	6	/
23	Apigenin 7-O-β-D-glucuronide	15.04	446.0833	447.0906	445.0753	4	2
24	Prunetin	15.97	284.0672	285.0745	283.0598	4	1
25	Baicalein	17.91	270.0517	271.0590	269.0508	4	3
26	Norwogonin	17.91	270.0517	271.0590	269.0508	4	3
27	Luteolin/Kaempferol	17.91	286.0463	287.0535	285.0387	5	2
28	Wogonin	20.67	284.0667	285.0740	283.0589	6	1
29	3',4',5'-O-trimethyltricetin	20.70	344.0891	345.0964	343.0816	1	1
30	Bis(2-ethylhexyl) phthalate	21.62	390.2748	391.2821	/	6	/
31	Dihydrowogonin	21.89	286.0835	287.0908	285.0764	2	3
32	Obacunone	23.55	454.1985	455.2058	/	1	/

“*/”, Note detected*.

**Table 2:** The quantification of 13 reference compounds in the extract of HLJDD (*n* = 6).

**Compound**	**Linear range (μg·mL^−1^)**	**Regression equation**	**Correlation coefficient, *R*^2^**	**Contents (mg·g^−1^)**
Geniposidic acid	0.21–209.23	Y = 2^*^10^−5^ X +0.604	0.9998	1.02
Chlorogenic acid	0.30–300.48	Y = 7^*^10^−6^ X −4.871	0.9999	4.14
Geniposide	0.23–234.38	Y = 4^*^10^6^ X −2.392	0.9997	1.18
Phellodendrine	0.10–103.67	Y = 2.3103^*^ X −3.006	0.9995	0.64
Magnoflorine	0.13–132.21	Y = 1^*^10^−5^ X −6.061	0.9996	14.08
Tetrahydroxpalmatine	0.26–256.91	Y = 2^*^10^−7^ X −2.247	0.9993	0.48
Coptisine	0.14–139.72	Y = 4^*^10^−7^ X −2.891	0.9993	49.92
Jatrorrhizine	0.12–117.19	Y = 2^*^10^−7^ X −1.789	0.9997	30.31
baicalin	0.26–262.17	Y = 2^*^10^−6^ X −3.208	0.9992	156.42
Palmatine	0.13–131.46	Y = 2^*^10^−7^ X −1.285	0.9994	20.13
Berberine	0.14–139.73	Y = 2^*^10^−7^ X −1.684	0.9994	142.80
Baicalein	0.08–81.13	Y = 1^*^10^−6^ X −0.628	0.9992	89.85
Wogonin	0.24–243.08	Y = 8^*^10^−7^ X −6.764	0.9997	1.21

### Urinary metabolites identification

**Figure 3:**
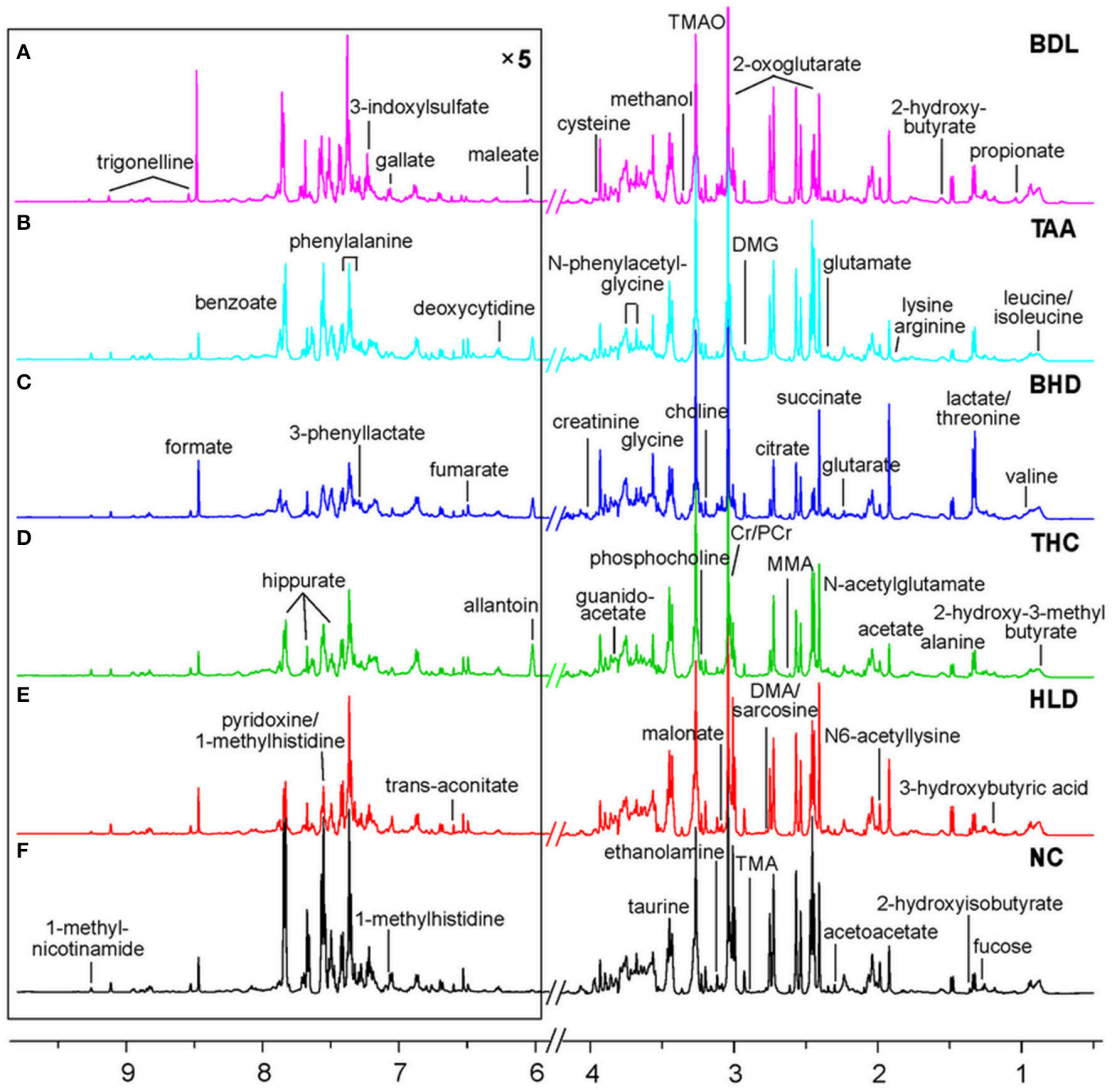
Typical 500 MHz 1H NMR spectra for urine of two cholestatic injuries model groups BDL (**A**, bile duct ligation) and TAA (**B**, intoxicated with thioacetamide), two HLJDD treatment groups BHD (**C**, on BDL model) and THC (**D**, on TAA model), and two control groups HLD **(E)** and NC **(F)** with metabolites assigned. MMA, methylamine; DMA, dimethyl amine; TMA, trimethylamine; TMAO, trimethylamine N-oxide; DMG, dimethyl glycine; Cr, creatine; PCr, phosphocreatine.

A combination of statistical total correlation spectroscopy, ^1^H-^1^H total correlation spectroscopy and ^1^H-^13^C heteronuclear single-quantum correlation was used to assign metabolites in urine, in a manner the same as before (Wei et al., 2014a,b). As a result of the joined effort of these techniques, 58 metabolites were identified and assigned (Table S1 and Figure 3).

### Comparison of two cholestatic injuries and the effects of HLJDD

Direct comparison of the two models was made by OPLS-DA analysis of urinary 1H NMR data of TAA, BDL, and NC groups. The BDL group dominated the variations, being far away from both the NC and TAA groups. NC and TAA groups, on the other hand, could only be marginally differentiated along the vertical axis. In consistent with the pathological observations, the score and the trajectory plots (Figures S1a,b) suggested that bile duct ligation induced mechanical cholestatic injuries was severer than thioacetamide induced metabolic cholestatic injuries, which might due to the persistent obstruction of bile duct in BDL model versus administration of thioacetamide only once in TAA model. Color-coded loading plot (Figure S1c) provided a direct visualization of the contribution of variables to group separation. Compared with TAA and NC groups, BDL group exhibited significantly increased levels of branched-chain amino acids (BCAAs: leucine, isoleucine and valine), lysine/arginine, phosphocholine, glycine and N-phenylacetyl-glycine, and strikingly decreased levels of citrate, 2-oxoglutarate (2-OG), fumarate and hippurate.

**Figure 4:**
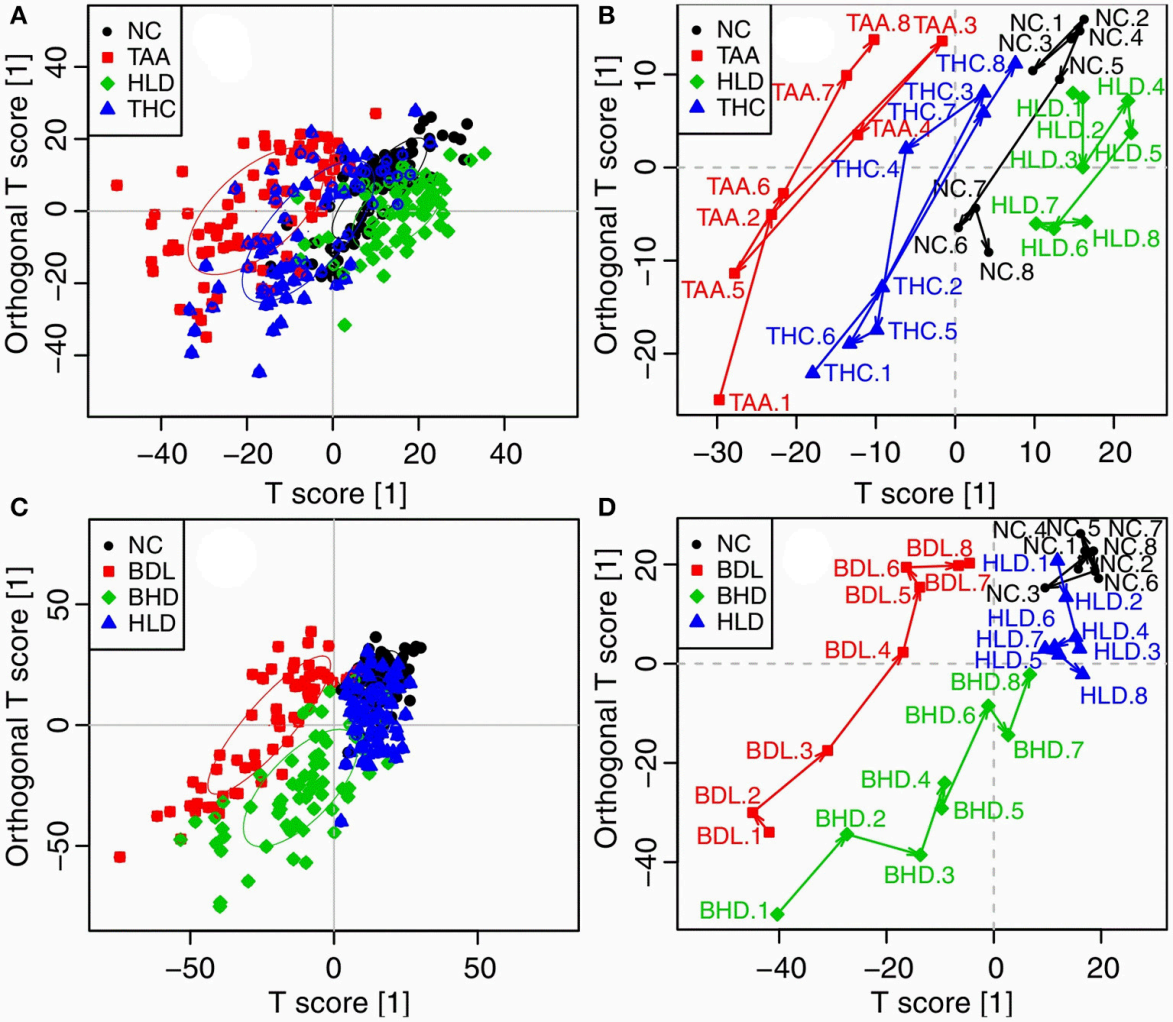
OPLS-DA analysis of 1H NMR data in urine for normal control rats (NC), healthy rats administrated with HLJDD (HLD), cholestatic injury rats induced by thioacetamide (TAA) and treated with HLJDD (THC) **(A,B)**, and NC, HLD, cholestatic injury rats induced by bile duct ligation (BDL) and treated with HLJDD (BHD) **(C,D)**. **(A,B)** scores plots; **(C,D)** mean trajectory plots.

The two cholestatic injuries and the treatment effects of HLJDD were compared according to the metabolomics profiles of ^1^H NMR data for HLD, NC, TAA, and THC groups (TAA model) and HLD, NC, BDL and BHD groups (BDL model). In the OPLS-DA score (Figure 4A) and trajectory plots (Figure 4B) for TAA model, the four groups formed three distinct clusters along the horizontal axis (T1 score): NC and HLD to the right, TAA to the left, and THC in the middle. TAA and THC groups showed a zigzag trajectory: being furthest away from the NC and HLD groups at T1, then moving back gradually till T4; from T5 and T6, moving toward the NC and HLD groups. The metabolomic profiles of groups in the BDL model resembled those in the TAA model (Figure 4C). However, the trajectories for the BDL and BHD groups (Figure 4D) showed trends different from those for the TAA and THC groups: being the furthest away from the NC and HLD groups at T1 and then moving steadily toward them. The NC and HLD rats could be differentiated along the vertical axis of corresponding score plots in both models. Therefore, the horizontal axis represented pathological changes in the two models; and the vertical axis reflected variations irrelevant to the effects of the two models, e.g., the effects of HLJDD on healthy rats.

### Influence of HLJDD on healthy rats

**Figure 5:**
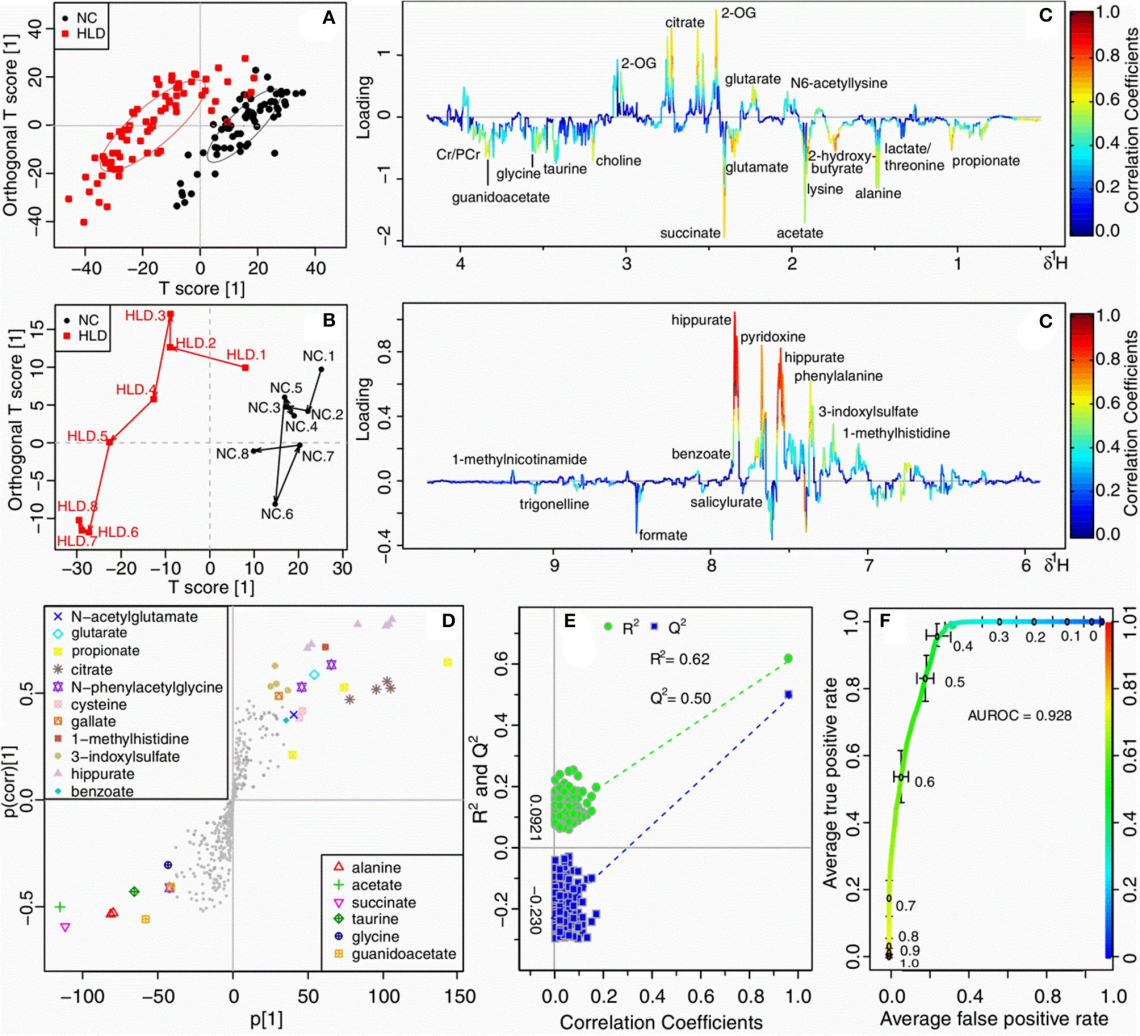
OPLS-DA analysis of urinary metabolomic profiles of normal control rats (NC) and healthy rats administrated with HLJDD (HLD). **(A)** scores plots; **(B)** mean trajectory plots; **(C)** loading plots color-coded according to the absolute value of correlation coefficients. 2-OG, 2-oxoglutarate; Cr, creatine; PCr, phosphocreatine; **(D)** S-plots; **(E)** scatter plots of statistical validation obtained by permutation test, with *R*^2^ and *Q*^2^ values in the vertical axis, the correlation coefficients in the horizontal axis, and the ordinary least squares (OLS) line for the regression of *R*^2^ and *Q*^2^ on the correlation coefficients, intercepts: *R*^2^ = (0.0, 0.0921), *Q*^2^ = (0.0, −0.230); **(F)** receiver operating characteristic (ROC) curves of classifier performance of OPLS-DA models, the x-axis denoting the false positive rate, the y-axis the true positive rate.

**Figure 6:**
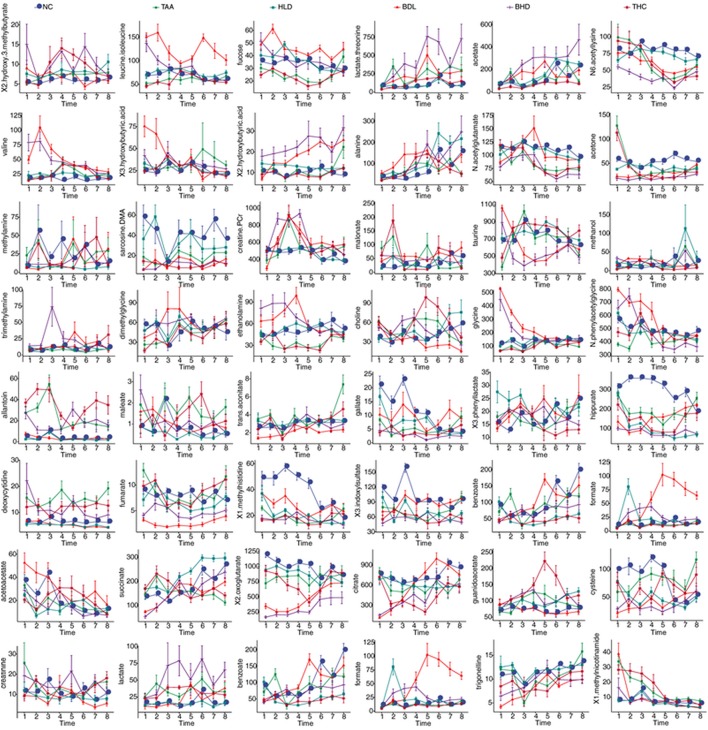
Lattice plots of metabolites levels in urine for two model groups TAA (intoxicated with thioacetamide) and BDL (bile duct ligation), two HLJDD treatment groups THC (on TAA model) and BHD (on BDL model), and two control groups NC and HLD. The horizontal axis denoted the time points and the vertical axis denoted the integrated areas of the metabolites.

An OPLS-DA analysis was performed on HLD and NC groups of all time-points to explore the metabolic alterations of HLJDD on healthy rats. The score (Figure 5A) and the mean trajectory plots (Figure 5B) showed a good separation between the two groups. In the loading plot (Figure 5C) and S-plot (Figure 5D), HLD group showed significantly increased levels of propionate, 2-hydroxybutyrate (2-HB), lysine/arginine, glutamate, succinate, choline and guanidoacetate, and noticeably decreased levels of citrate, 2-OG, pyridoxine, phenylalanine and hippurate. The OPLS-DA model constructed for HLD and NC groups demonstrated a good prediction with *R*^2^ at 0.620 and *Q*^2^ at 0.500 (Figure 5E) and a favorable discrimination with AUROC of 0.928 (Figure 5F). The variations of metabolites in HLD group were shown in lattice plots (Figure 6), and their levels relative to the NC group were presented in color-coded table (Table 3). Compared with NC, levels of 2-HB, glutamate and glycine were noticeably increased in the urine of HLD group, while concentration of pyridoxine, benzoate, hippurate, and 3-indoxylsulfate were significantly decreased.

**Table 3:**
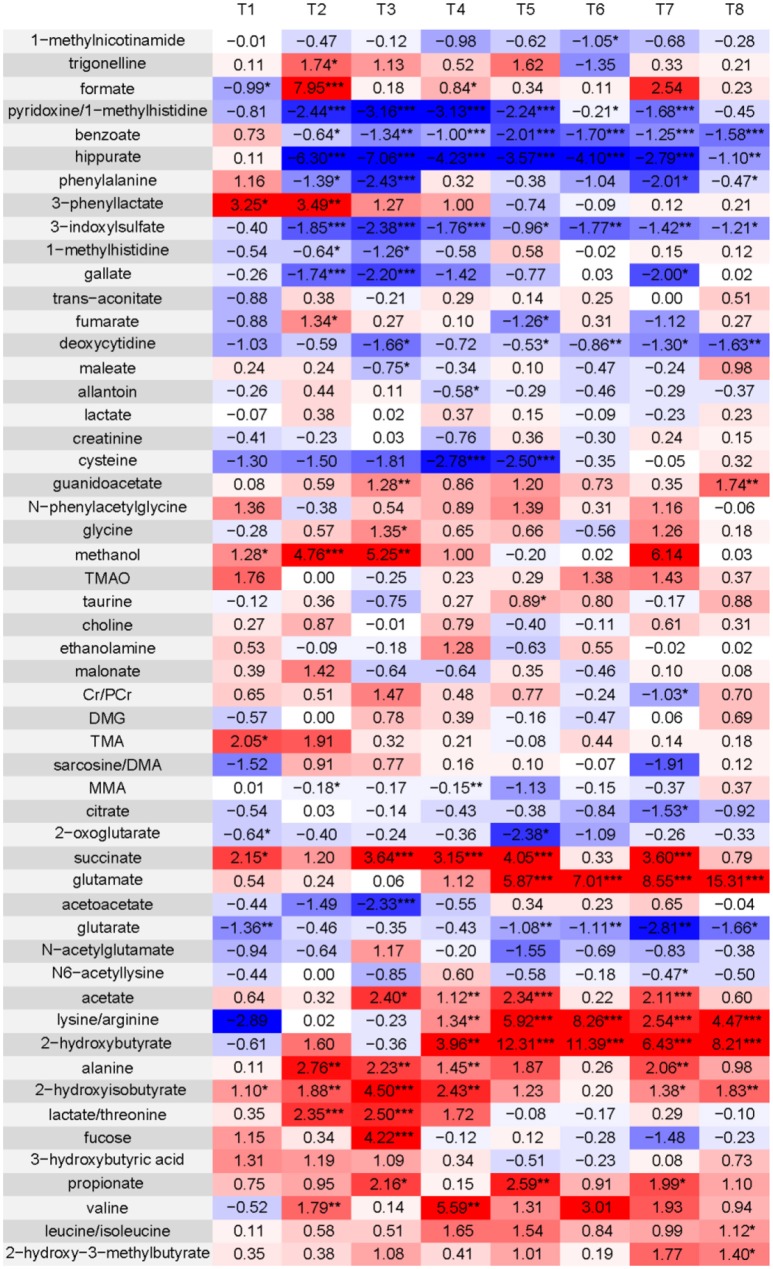
Levels of integrated areas of metabolites in HLJDD administrated healthy rats (HLD) relative to normal control rats (NC) as depicted by z-scores (z-scores are colored for easy interpretation).

z-score = (mean_HLD_−mean_NC_)/SD_NC._

^*^p < 0.05, ^**^p < 0.01, ^***^p < 0.001: compare to NC group.

*Color code 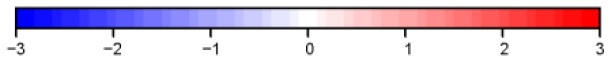
*.

### Treatment of HLJDD on thioacetamide induced cholestatic rats

**Figure 7:**
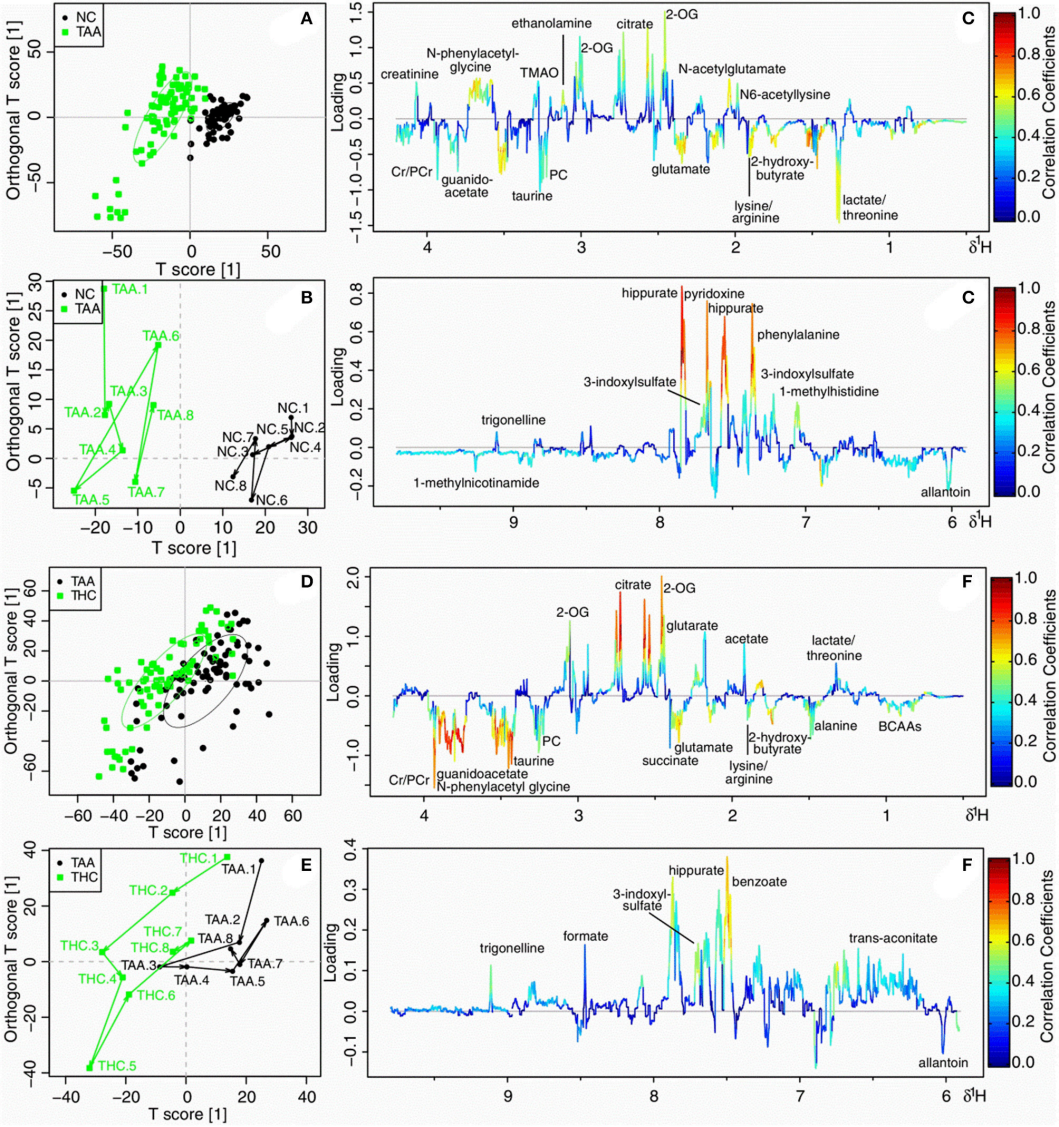
OPLS-DA analysis of 1H NMR data in urine for normal control rats (NC) vs. cholestatic injury rats induced by thioacetamide (TAA) **(A–C)**, and TAA rats vs. HLJDD treated TAA rats (THC) **(D–F)**. **(A,D)** scores plots; **(B,E)** mean trajectory plots; **(C,F)** loading plots color-coded according to the absolute value of correlation coefficients. BCAAs: branched-chain amino acids, leucine, isoleucine and valine; 2-OG: 2-oxoglutarate; PC: Phosphocholine; TMAO: trimethylamine N-oxide; Cr: creatine; PCr: phosphocreatine.

NMR data of TAA vs. NC, and THC vs. TAA groups throughout the experiment were subjected to OPLS-DA analysis to unravel metabolic outcomes associated with cholestatic injuries induced by thioacetamide, and to find out metabolites that were directly related to the treatment effects of HLJDD on TAA model. TAA and NC groups showed good separation in score (Figure 7A) and trajectory plots (Figure 7B), with favorable *R*^2^ of 0.510 and *Q*^2^ of 0.450 (Figure S2a), and AUROC of 0.947 (Figure S3a), demonstrating a good distinction of TAA from NC group. THC was partly separated from TAA in the score (Figure 7D) and trajectory plots (Figure 7E), together with unfavorable values of *Q*^2^ (0.035, Figure S2b) and AUROC (0.763, Figure S3b), implying an unobvious metabolite change to TAA rats on the treatment of HLJDD. The loading plot (Figure 7C) revealed markedly increased levels of lactate/threonine, lysine/arginine and glutamate, and noticeably reduced levels of N-acetylglutamate, N-phenylacetyl-glycine, pyridoxine, phenylalanine and hippurate in TAA group, as compared with NC rats. Compared with TAA rats, THC rats exhibited increased levels of lysine/arginine, glutamate, phosphocholine, taurine, guanidoacetate, N-phenylacetyl-glycine, and Cr/PCr, and decreased levels of glutarate, 2-OG, citrate, benzoate, hippurate, 3-indoxylsulfate and trigonelline (Figure 7F).

However, most of these alterations couldn't support the beneficial effects of HLJDD on TAA model; neither did the univariate analysis of metabolites in THC group relative to NC group. To deduct the effect of HLJDD on metabolic status of healthy rats, metabolites of THC group were normalized by HLD group. Levels of metabolites in THC group relative to HLD group showed great difference to those in TAA group relative to NC group, especially those of glutamate, glycine, 2-HB, benzoate, pyridine and N-acetylglutamate. Variations of metabolites in TAA group were visualized relative to NC rats (Table S2). Throughout the whole experiment, significant high levels of allantoin (*P* < 0.01) were observed in TAA rats. Glutamate and 2-HB were observed increased in THC group relative to NC group (Table S3); however, their levels in THC group were decreased from T5 to T8 relative to HLD group (Table S4). N-acetylglutamate and hippurate were decreased in TAA rats from T2 to T7, which were significantly increased in THC group relative to HLD group, suggestive the protective effect of HLJDD on the damaged liver mitochondria.

### Treatment of HLJDD on bile duct ligated cholestatic rats

**Figure 8:**
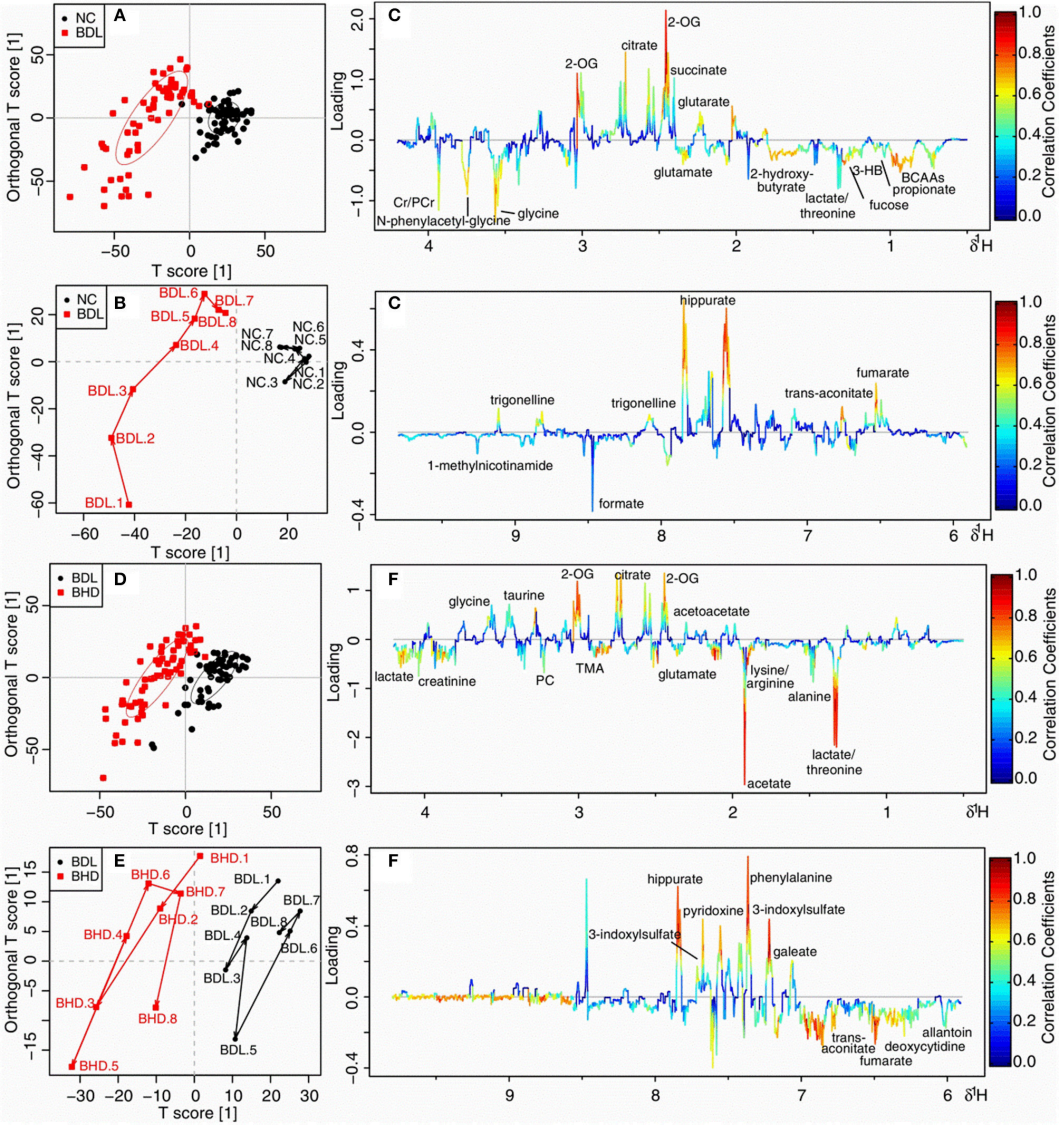
OPLS-DA analysis of 1H NMR data in urine for normal control rats (NC) vs. cholestic injury rats induced by bile duct ligated (BDL) **(A–C)**, and BDL vs. HLJDD treated BDL rats (BHD) **(D–F)**. **(A,D)** scores plots; **(B,E)** mean trajectory plots; **(C,F)** loading plots color-coded according to the absolute value of correlation coefficients. BCAAs, branched-chain amino acids, leucine, isoleucine and valine; 3-HB, 3-hydroxybutyrate; 2-OG, 2-oxoglutarate; PC, Phosphocholine; TMA, trimethylamine; Cr, creatine; PCr, phosphocreatine.

NMR data of BDL vs. NC, and BHD vs. BDL groups from T1 to T8 were subjected to OPLS-DA analysis to explore the metabolic feature of cholestatic injuries induced by bile duct ligation and the treatment effects of HLJDD on it. Clear separation was achieved between the two groups in both constructed OPLS-DA models in score (Figures 8A,D) and trajectory plots (Figures 8B,E). The favorable model parameters showcased a good performance and prediction ability of the constructed model (*R*^2^ = 0.640, *Q*^2^ = 0.590 in Figure S2c, and AUROC = 0.942 in Figure S3c for BDL vs. NC groups; *R*^2^ = 0.470, *Q*^2^ = 0.420 in Figure S2d, and AUROC = 0.986 in Figure S3d for BHD vs. BDL groups). Compared with NC rats, BDL rats exhibited significantly increased levels of BCAAs, fucose, lysine/arginine, glutamate, glycine, N-phenylacetyl-glycine and creatine/phosphocreatine (Cr/PCr), and decreased levels of glutarate, 2-OG, fumarate, hippurate and trigonelline (Figure 8C). BHD rats showed increased levels of lactate/threonine, lysine/arginine, acetate and fumarate, and decreased levels of 2-OG, citrate, 3-indoxylsulfate, phenylalanine, pyridoxine and hippurate as compared with the NC group (Figure 8F).

**Figure 9:**
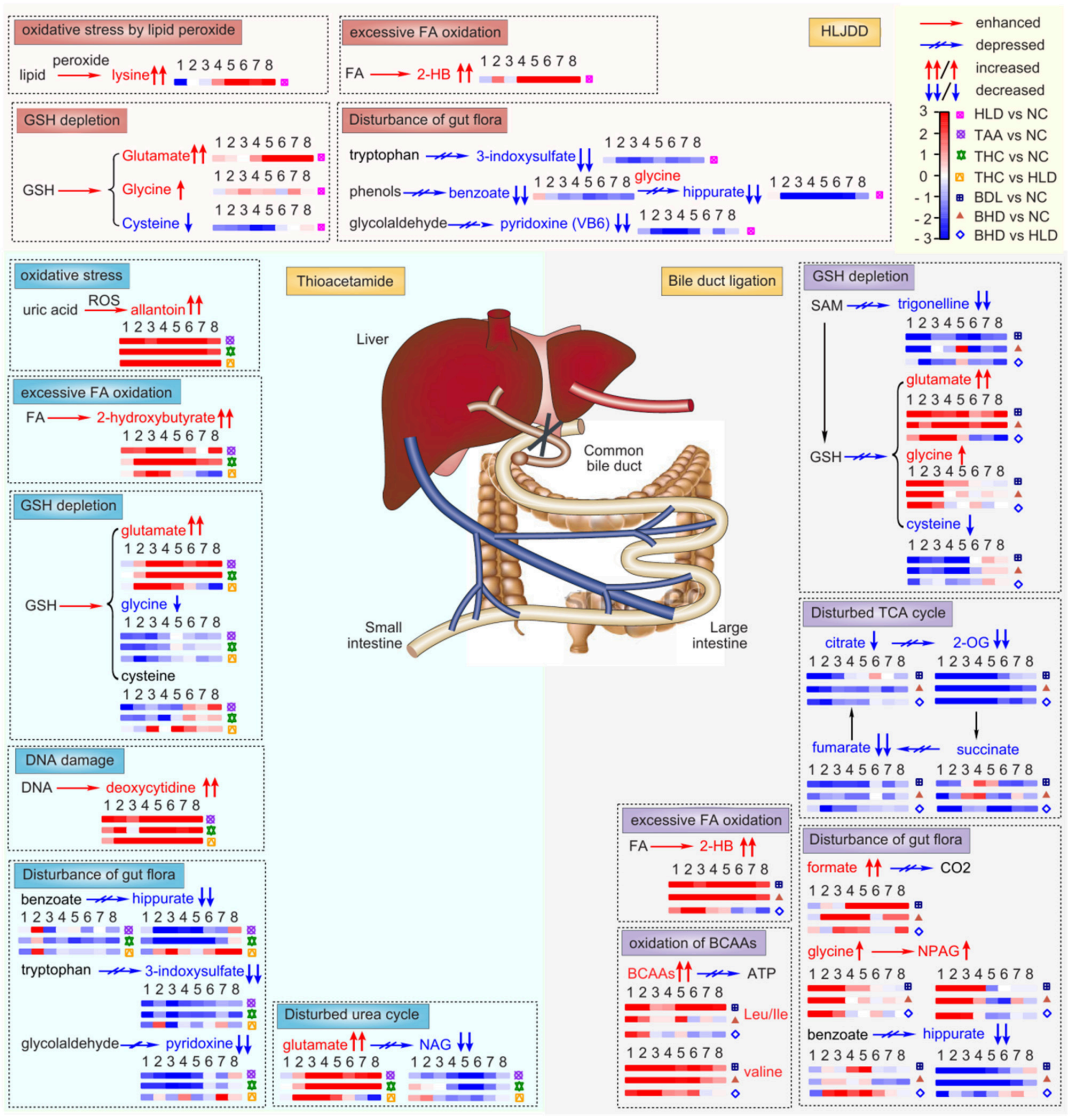
Noticeably altered metabolites in two cholestatic injury model groups TAA (intoxicated with thioacetamide) and BDL (bile duct ligation), two HLJDD treatment groups THC (on TAA model) and BHD (on BDL model), and HLJDD administrated healthy rats (HLD). NC, normal control group; FA, fatty acid; 2-HB, 2-hydroxybutyrate; GSH, glutathione; ROS, reactive oxygen species; BCAAs, branch-chained amino acids, leucine, isoleucine and valine; ATP, triphosphate adenosine; SAM, S-adenosyl-methionine; Kreb's cycle, tricarboxylic acid cycle; 2-OG, 2-oxoglutarate; NAG, N-acetylglutamate; NAPG, N-phenylacetyl-glycine. ↑↑/↓↓ and ↑/↓ stands for significantly and partly altered (increased or decreased), respectively.

Color-coded table (Table S5) was used to visualize important variations of metabolites in BDL group relative to NC group. Glutamate and 2-HB showed noticeable sustained increase from T1 to T8 in BDL rats. Their levels were still increased in BHD group relative to NC group (Table S6), and were decreased relative to HLD group (Table S7). Glycine showed higher levels in BDL rats than that in NC rats throughout the experiment but gradually declined from T1 to T8. The high levels of glycine in BDL group from T4 to T5 were decreased in BHD group relative to HLD group (Figure 9). Trigonelline showed decreased level in BDL group throughout the whole experiments, which exhibited increased level in BHD group at T5. Similar to trigonelline, 2-OG, hippurate and fumarate were also decreased in BDL rats. Increased hippurate and decreased formate and N-phenylacetyl-glycine were found in BHD group relative to HLD group.

## Discussion

The extract was analyzed by UPLC-LTQ-Orbitrap-MS to successfully identify the major chemical component in HLJDD as phthalate, iridoid and its glycosides, flavonoids and alkaloids. Among the quantified compounds in HLJDD extract, baicalin, baicalein and berberine were exhibited to be the most abundant (Table S2), which was in accordance with previously reported (Xu et al., 2017; Zhang et al., 2017a). As the represent hepatic protective chemical components of *Radix Scutellariae* and HLJDD, baicalin was reported to ameliorate experimental cholestatic liver fibrosis in mice by modulation of oxidative stress, inflammation, and NRF2 transcription factor (Shen et al., 2017), attenuate non-alcoholic steatohepatitis by suppressing key regulators of lipid metabolism, inflammation and fibrosis in mice (Zhang et al., 2018), attenuate the liver injury induced by alcohol through modulation of hepatic oxidative stress, inflammation and sonic hedgehog pathway (Wang H. et al., 2016), and prevent against acetaminophen-induced liver injury via ERK signaling pathway (Liao et al., 2017). Moreover, it showed that baicalein and baicalin could alleviate acetaminophen-induced liver injury by activating Nrf2 antioxidative pathway (Shi et al., 2018). As the major and represent alkaloid of both *Rhizoma Coptidis, Cortex Phellodendri Chinensis*, and HLJDD, berberine could inhibit the liver fibrogenesis both *in vivo* and *in vitro* (Wang N. et al., 2016), alert hepatoprotective effect against methotrexate induced liver toxicity in rats (Mehrzadi et al., 2018), and promote male-specific expression of a bile acid uptake transporter to ameliorate liver cholestasis by inactivation of signal transducer and activator of transcription 5 signaling (Bu et al., 2017). HLJDD and its major representative components of baicalin, baicalein and berberine could protect against various hepatic injuries by ameliorating metabolic disturbances and improving hepatic function.

### Excessive oxidative stress

The two acute cholestasis model shared common excessive fatty acid oxidation, as revealed by the notably elevated levels of lysine and 2-HB both in the urine of TAA and BDL rats. Lysine and 2-HB were indicative of oxidative stress induced by lipid peroxide (O'Doherty et al., 2014) and by excessive fatty acid oxidation (Gall et al., 2010), respectively. Reactive oxygen species (ROS) facilitated excessive fatty acid oxidation, as evidenced by the increased levels of 2-HB in TAA rats (Gall et al., 2010). As one of the representativ phenols and iridoid glycosides of HLJDD, chlorogenic acid and geniposide were reported to decrease the serum concentration of 2-HB (Ruan et al., 2014) and to confront glutamate-induced obese and exhibit antiobesity effects (Shi et al., 2011). The findings were consistent with the results of THC and BHD rats after administration with HLJDD in our study.

As a biomarker for oxidative stress, allantoin was originated from antioxidant uric acid in case of ROS (Yardim-Akaydin et al., 2004). Its elevated levels in TAA rats thus disclosed a rise in the production of endogenous ROS after thioacetamide intoxication, in consistent with previous report (Stanková et al., 2010). The levels of allantoin in the serum and urine were both increased in collagen-induced arthritis, which were decreased after supplementation with HLJDD and its combination formula with chemical constituents including 14% geniposide, 0.6% coptisine, 0.9% phellodendrine, 3% jatrorrhizine, 3.2% magnoflorine, 5.1% palmatine, 32% berberine, 26.1% baicalin, 2.6% chlorogenic acid, 1% crocin, 5.9% wogonoside, 3.6% baicalein, and 2.2% wogonin (Zhang et al., 2014).

### Glutathione depletion

Excessive oxidative stress in the two acute cholestasis model resulted in depletion of glutathione, as revealed by the notably elevated level of glutamate and disturbed contents of glycine, precursors of the most import natural antioxidant glutathione (GSH), implying the depletion of GSH (Choucha Snouber et al., 2013). Chlorogenic acid was reported to increase serum concentrations of glycine and hepatic concentrations of glutathione (Ruan et al., 2014). Therefore, comes naturally for the decrease of glutamate in BHD and THC rats after administration of HLJDD since that HLJDD is rich in chlorogenic acid.

Trigonelline is the methylation product of nicotinic acid in the demethylation process from S-adenosyl-methionine (SAM) to S-adenosyl-homocysteine (SAH) (Sun et al., 2008). S-adenosyl-homocysteine after hydrolysis afford homocysteine, which could be converted eventually into glutathione. Therefore, the increase of trigonelline in BHD group suggested an increased conversion from S-adenosyl-methionine to S-adenosyl-homocysteine, thus a regeneration of glutathione to regain the balance of redox system. Trigonelline was reported to protect the liver-kidney functions of diabetic rats efficiently (Hamden et al., 2013), thus increased trigonelline in BHD group suggested regained function of livers and kidneys of BDL rats, in consistent with the histopathological observation.

### DNA damage

ROS could also led to mitochondrial injury and even cell death of hepatocytes (Stanková et al., 2010). Bilirubin in dead hepatocytes entered blood, exhibiting symptoms of jaundice in TAA rats, as evidenced by the obviously consistent high levels of deoxycytidine, biomarker for jaundice syndrome (Wang X. et al., 2012), in TAA rats. Deoxycytidine is a composition of DNA, thus its continuous increase also suggested DNA damage in TAA rats, which has also been observed in the toxicity of thioacetamide S-oxides, metabolites of thioacetamide (Lozano et al., 2014).

### Disturbed urea cycle

ROS induced injury of mitochondria in livers, led to the obstacle of urea cycle as evidenced by decreased level of N-acetylglutamate from T2 to T7 in TAA rats (Ishihara et al., 2006), which was increased after treatment with HLJDD. N-acetylglutamate is the most important cofactor in the control of ureagenesis in the mitochondria of liver (Tuchman et al., 2008), thus its increase in THC group relative to HLD group demonstrated that HLJDD could reverse the disturbed urea cycle in cholestasis TAA model. Synthesized from glutamate, the decrease of N-acetylglutamate has a direct association with the increased level of glutamate.

### Disturbance of gut flora

As an antioxidant produced by the intestinal bacteria (Cornelli et al., 2010), the level of Pyridoxine (ca. VB6) was decreased in HLD group compared with NC rats, which suggested a dysfunction of gut flora that also evidenced by significantly decreased levels of benzoate, hippurate, and 3-indoxylsulfate (mammalian-microbial co-metabolites). Their decrease was consistent with oral administration of antibiotics of penicillin and streptomycin sulfate (Swann et al., 2011), therefore, comes naturally for their decrease in HLD rats since that HLJDD is rich in antibacterial berberine and other principles (Lewis and Ausubel, 2006). Therefore, it is strongly recommended to avoid taking any natural or artifact bacteriostatic agents including TCM such as HLJDD unless it is necessary.

Hippurate was generated in rat liver mitochondria by the conjugation of glycine with benzoate that was synthesized by bacterial action in gut. The decreased hippurate in BDL groups thus indicated disturbed gut flora, also supported by increased levels of two other gut microbial-host co-metabolites such as formate and N-phenylacetyl-glycine (Dong et al., 2012). The bile in cholestatic BDL rats couldn't flow to duodenum, resulting in alteration of microenvironment of gut and dysfunction of the gut flora. Gut microbiota was able to metabolize polyphenols, such as chlorogenic acid, into more absorbable compounds such as benzoic acid which was then detoxied to form hippurate (Claus et al., 2008). After administration of HLJDD, increased hippurate and decreased formate and N-phenylacetyl-glycine in BHD group relative to HLD group were observed, in accordance with previously reported, suggesting that HLJDD and its main principle chlorogenic acid could regain the unbalanced gut flora.

### Dysfunction of energy metabolism

As the intermediates of Kreb's cycle, their significant decrease and slight decrease of citrate and succinate in BDL rats demonstrated a limited supply of substrates to the Kreb's cycle and thus an insufficient energy production. The energy crisis was even aggravated as evidenced by the increased level of BCAAs in BDL rats from T1 to T8. BCAAs play essential role in energy supply, especially for muscles and other organs by their oxidation (Choudry et al., 2006). The accumulation of BCAAs in BDL rats thus suggested an inhibited oxidation to compensate energy insufficiency. The increase of BCAAs in BDL group indicated renal injury, in consistent with those of nephrotoxicity study induced by D-serine (Williams and Lock, 2005), as evidenced by the observed severe pathological alterations in kidneys. The increased levels of fumarate and decreased levels of BCAAs in BHD group relative to HLD group suggested that HLJDD could regain the energy balance by regulating disturbed Kreb's cycle and inhibited BCAAs oxidation, in consistent with the protection of baicalin on liposaccharide induced damage (Liao et al., 2016b).

## Conclusion

The two cholestatic injury models induced by thioacetamide and bile duct ligation, and the treatment effect of HLJDD were investigated and compared using an NMR-based metabolomic approach, complemented with histopathological inspection. The common metabolic features in both cholestatic models were excessive fatty acid oxidation, insufficient glutathione regeneration and disturbed gut flora. Model specific metabolic characteristics were inhibited urea cycle and DNA damage in TAA model, and perturbed Kreb's cycle and inhibited BCAAs oxidation in BDL model. Rich in various bioactive polyphenols, flavonoids, iridoid glycosides and alkaloids, such as chlorogenic acid, baicalin, geniposide and berberine, HLJDD could regain the balance of the disturbed metabolic status common in the two cholestasis injuries with good treatment effects, e.g. unbalanced redox system and disturbed gut flora; and perturbed urea cycle in TAA model, and disturbed Kreb's cycle and oxidation of BCAAs) in BDL model, respectively (Figure 9).

## Author contributions

D-DW made substantial contributions to the conception, design of experiment, data acquisition and analysis, interpretation of data, and agreement to be accountable for all aspects of the work in ensuring that questions related to the accuracy or integrity of any part of the work are appropriately investigated and resolved. J-SW made substantial contributions to the analysis of the NMR-based metabolomics data, drafting the work, revising it critically for important intellectual content, and agreement to be accountable for all aspects of the work in ensuring that questions related to the accuracy or integrity of any part of the work are appropriately investigated and resolved. J-AD and L-YK made substantial contributions to the approval of the version to be published, and agreement to be accountable for all aspects of the work in ensuring that questions related to the accuracy or integrity of any part of the work are appropriately investigated and resolved.

### Conflict of interest statement

The authors declare that the research was conducted in the absence of any commercial or financial relationships that could be construed as a potential conflict of interest. The reviewer SC and handling Editor declared their shared affiliation.

## Abbreviations

2-OG: 2-oxoglutarate
2-HB: 2-hydroxybutyrate
AUROC: area under the receiver operating characteristic plot
BDL: bile duct ligation
BHD: bile duct ligation animals treated with Huang-Lian-Jie-Du-Decoction
BCAAs: branched-chain amino acids
Cr: creatine
GC-MS: gas chromatography-mass spectrometry
*R*^2^: goodness-of-fit
*Q*^2^: goodness of prediction
HE: hematoxylin & eosin
HLJDD: Huang-Lian-Jie-Du-Decoction
HMDB: Human Metabolome Database
LC-MS: liquid chromatography mass spectrometry
HLD: normal animals administrated with Huang-Lian-Jie-Du-Decoction
NC: normal control
NMR: nuclear magnetic resonance
OPLS-DA: orthogonal partial least squares-discriminant analysis
PCr: phosphocreatine
ROC: receiver operating characteristic
TAA: thioacetamide-intoxicated
THC: thioacetamide-intoxicated animals treated with Huang-Lian-Jie-Du-Decoction
TCM: Traditional Chinese medicine
UPLC: ultra-high pressure liquid chromatography.
