# Supplementary material for: Metabolomic Assessment of Acute Cholestatic Injuries Induced by Thioacetamide and by Bile Duct Ligation, and the Protective Effects of Huang-Lian-Jie-Du-Decoction
Source: Front Pharmacol. 2018 May 8;9:458. doi: 10.3389/fphar.2018.00458 (PMC5952270; doi:10.3389/fphar.2018.00458)
Supplement: Supplementary file 1 [file Data_Sheet_1.DOCX]

Supporting information

Metabolomic assessment of acute cholestatic injuries induced by thioacetamide and by bile duct ligation, and the protective effects of Huang-Lian-Jie-Du-Decoction

Dan-Dan Wei^1, 2^, Jun-Song Wang^3^, Jin-Ao Duan*^1^, Ling-Yi Kong*^2^

^1^Jiangsu Collaborative Innovation Center of Chinese Medicinal Resources Industrialization, National and Local Collaborative Engineering Center of Chinese Medicinal Resources Industrialization and Formulae Innovative Medicine, Nanjing University of Chinese Medicine, Nanjing 210023, China

^2^State Key Laboratory of Natural Medicines, Department of Natural Medicinal Chemistry, China Pharmaceutical University, 24 Tong Jia Xiang, Nanjing 210009, P. R. China

^3^Center for Molecular Metabolism, Nanjing University of Science and Technology, 200 Xiao Ling Wei, Nanjing, 210094, P. R. China

* Correspondence:

Prof. Jin-Ao Duan

Tel/Fax: +86 25 85811116; E-mail: dja@njutcm.edu.cn

Prof. Ling-Yi Kong

Tel/Fax: +86 25 8327 1405; [cpu_lykong@126.com](mailto:cpu_lykong@126.com)

^#^These authors contributed equally to the manuscript.

Figure caption


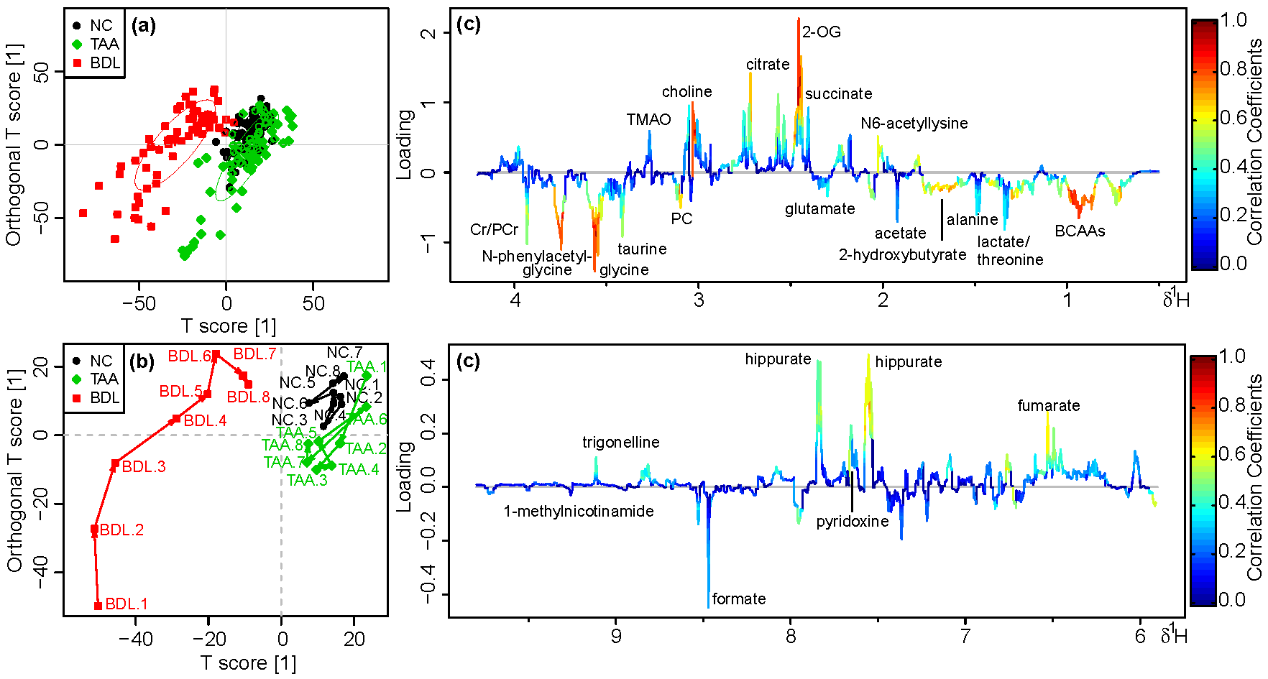


Fig. S1 OPLS-DA analysis of ^1^H NMR data in urine for normal control rats (NC), cholestatic injuries rats induced by thioacetamide (TAA) and by bile duct ligation (BDL). (a) : scores plots; (b): mean trajectory plots; (c): loading plots color-coded according to the absolute value of correlation coefficients. BCAAs: branched-chain amino acids, leucine, isoleucine and valine; 2-OG: 2-oxoglutarate; PC: Phosphocholine; TMAO: trimethylamine N-oxide; Cr: creatine; PCr: phosphocreatine.


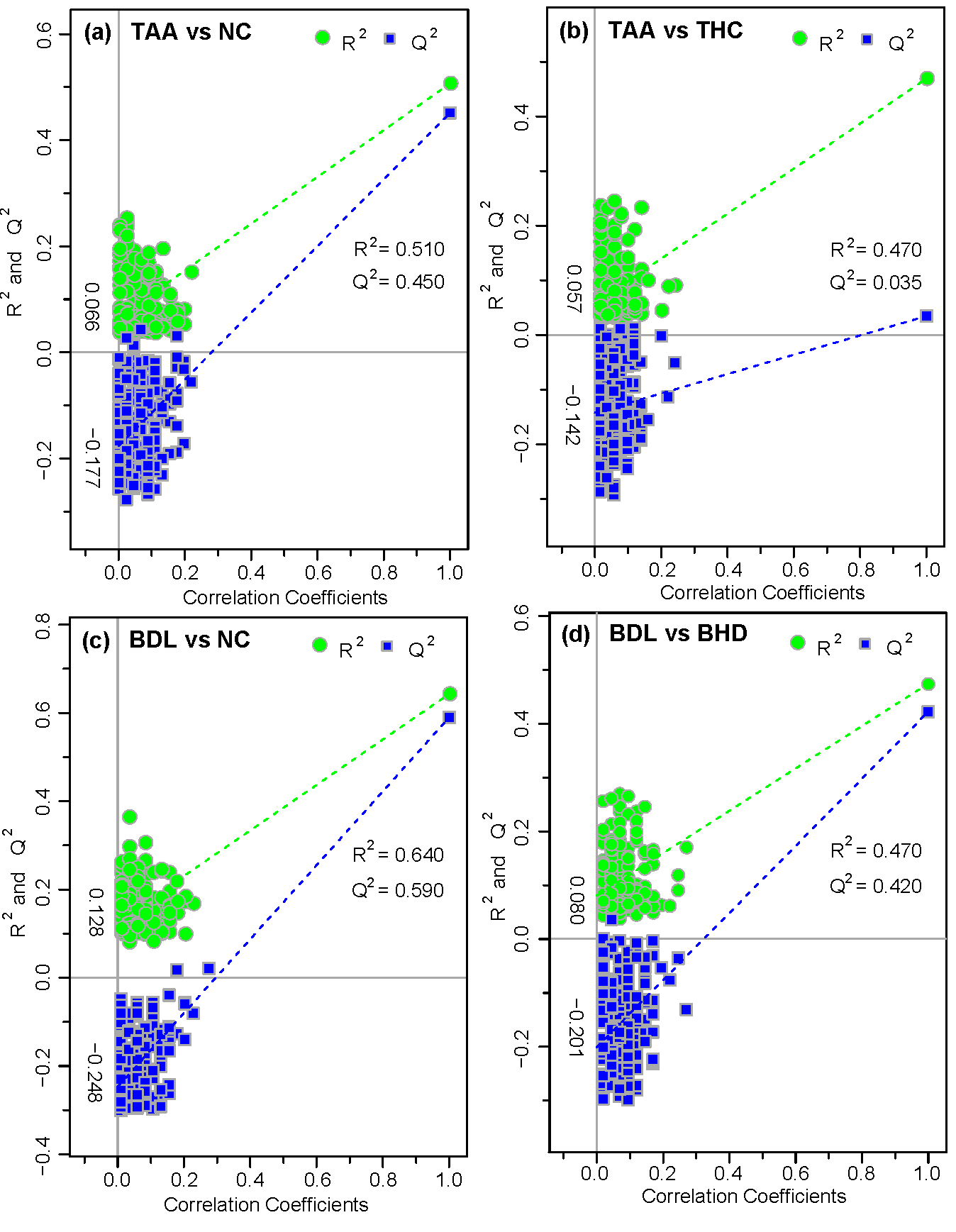
Fig. S2 Scatter plots of statistical validation of OPLS-DA analysis of normal control rats (NC) versus cholestatic injury rats induced by thioacetamide (TAA) (a), TAA rats versus HLJDD treated TAA rats (THC) (b), NC versus cholestatic injury rats induced by bile duct ligation (BDL) (c) and BDL versus HLJDD treated BDL rats (BHD) (d) obtained by permutation test, the horizontal axis showing the correlation between the permuted and actual data, the vertical axis representing the value of R^2^ and Q^2^. Intercepts: R^2^ = (0.0, 0.066), Q^2^ = (0.0, -0.177) (a); R^2^ = (0.0, 0.057), Q^2^ = (0.0, -0.142) (b); R^2^ = (0.0, 0.128), Q^2^ = (0.0, -0.248) (c); R^2^ = (0.0, 0.080), Q^2^ = (0.0, -0.201) (d).


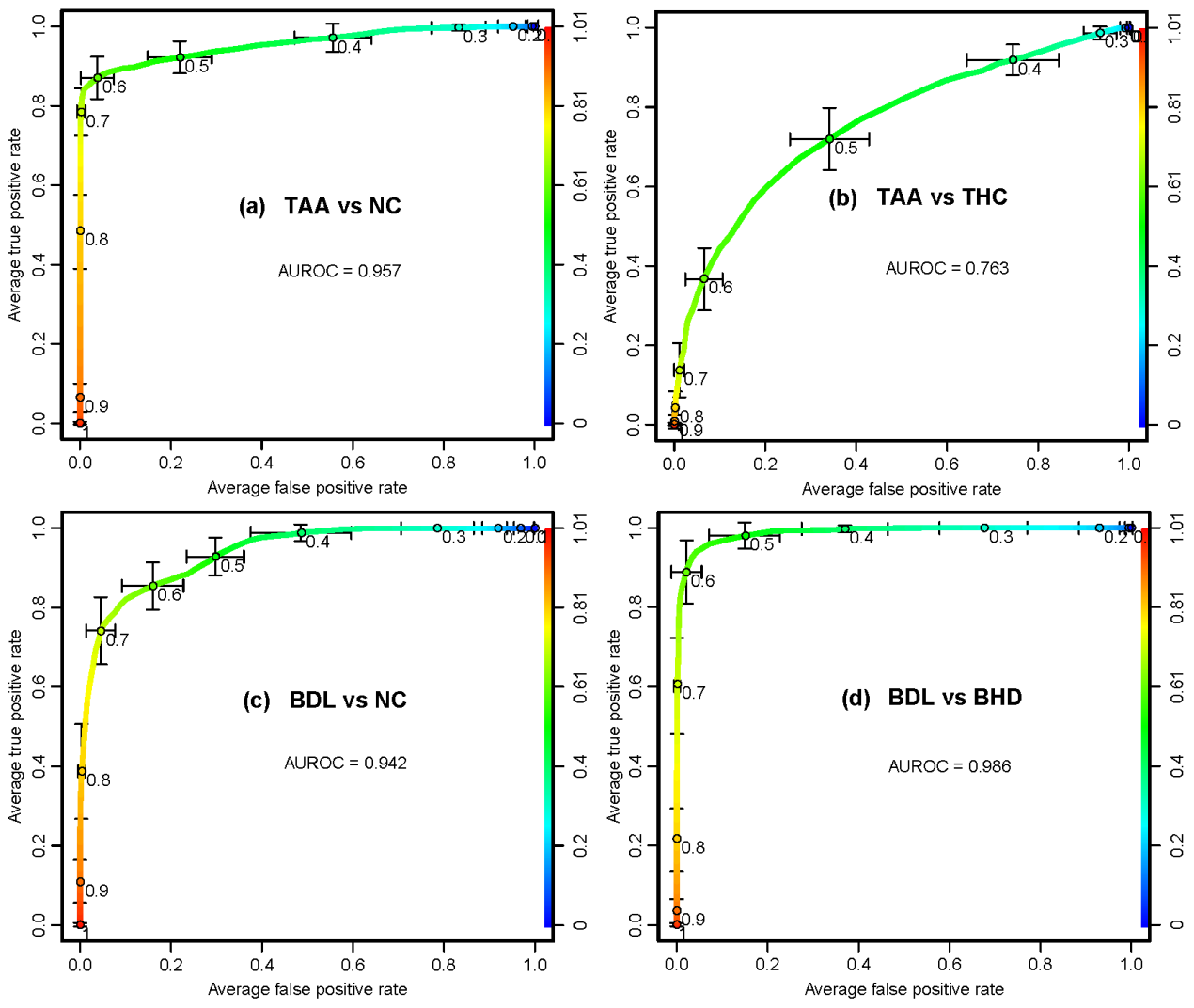


Fig. S3 Receiver operating characteristic (ROC) curves of classifier performance of OPLS-DA models of normal control rats (NC) versus cholestatic injury rats induced by thioacetamide (TAA) (a), TAA rats versus HLJDD treated TAA rats (THC) (b), NC versus cholestatic injury rats induced by bile duct ligation (BDL) (c) and BDL versus HLJDD treated BDL rats (BHD) (d), the x-axis denoting the false positive rate, the y-axis the true positive rate.

Tables legends:

Table S1 The assignment of metabolites in urine of NC, HLD, TAA, BDL, THC and BHD rats

| No. | Metabolite | Assignments | Chemical shift |
| --- | --- | --- | --- |
| 1 | 2-Hydroxy-3-methylbutyrate | γCH3, βCH, αCH | 0.83(d), 0.97(d), 2.02(m), 3.85(d) |
| 2 | Leucine | δCH3 , δCH3, γCH, αCH | 0.93(t), 0.95(t), 1.71(m), 3.74(m) |
| 3 | Isoleucine | δCH3 , γCH3 , αCH | 0.92(t), 1.0(d), 1.46(m) |
| 4 | Valine | γCH3, γCH3 | 0.98(d), 1.03(d), 2.26(m), 3.60(d) |
| 5 | Propionate | βCH3, αCH2 | 1.04(t), 2.17(q) |
| 6 | 3-Hydroxybutyrate | γCH3, βCH, αCH2 | 1.20(d), 2.31(m), 2.41(m), 4.16(m) |
| 7 | Fucose | εCH3, εCH, δCH, γCH, βCH, αCH | 1.21(d), 1.26(d), 3.45(m), 3.64(m), 3.79(m), 3.88(dd), 4.20(q), 4.58(d), 5.21(d) |
| 8 | Threonine | CH3, αCH, βCH | 1.33(d), 3.59(d), 4.26(m) |
| 9 | Lactate | CH3, CH | 1.32(d), 4.16(q) |
| 10 | 2-Hydroxyisobutyrate | αCH_3_ | 1.36(s) |
| 11 | Alanine | βCH_3_, αCH | 1.48(d), 3.78(q) |
| 12 | 2-Hydroxybutyrate | γCH_3_, βCH_2_, αCH | 0.90(t), 1.64(m), 1.74(m), 3.99(dd) |
| 13 | Lysine | δCH_2_ | 1.48(m), 1.73(m), 1.91(m), 3.03(t), 3.76(t) |
| 14 | Arginine | δCH_2_, γCH_2_, βCH_2_, αCH | 1.87(m), 1.90(m), 3.23(t), 3.76(t) |
| 15 | Acetate | CH_3_ | 1.92(s) |
| 16 | N6-Acetyllysine | CH_3_, εCH_2_, δCH_2_, γCH_2_, βCH_2_, αCH | 1.41(m), 1.56(m), 1.87(m), 1.97(s), 3.19(q), 3.74(t) |
| 17 | N-acetylglutamate | CH_3_, γCH_2_, βCH_2_, αCH | 1.87(m), 2.02(s), 2.05(m), 2.23(t), 4.10(m) |
| 18 | Glutarate | γCH_2_, βCH_2_, αCH_2_ | 1.78(m), 2.18(t) |
| 19 | Acetoacetate | γCH_3_, αCH_3_ | 2.30(s), 3.43(s) |
| 20 | Glutamate | βCH_2_, βCH_2_, γCH_2_, αCH | 2.10(m), 2.14(m), 2.34(m), 2.50(m), 3.77(t) |
| 21 | Succinate | CH_2_ | 2.41(s) |
| 22 | 2-Oxoglutarate | γCH_2_, βCH_2_ | 2.45(t), 3.00(t) |
| 23 | Citrate | 1/2CH_2_, 1/2CH_2_ | 2.67(d), 2.74(d) |
| 24 | Methylamine | CH_3_ | 2.61(s) |
| 25 | Sarcosine | CH_2_, N-CH_3_ | 2.72(s), 3.59(s) |
| 26 | Dimethylamine | CH_3_ | 2.71(s) |
| 27 | Trimethylamine | CH_3_ | 2.90(s) |
| 28 | Dimethylglycine | CH_3_, NH_2_ | 2.91(s), 3.71(s) |
| 29 | Creatine | CH_2_, N-CH_3_ | 3.04(s), 3.93(s) |
| 30 | Phosphocreatine | CH_2_, N-CH_3_ | 3.04(s), 3.93(s) |
| 31 | Malonate | CH_2_ | 3.09(s) |
| 32 | Ethanolamine | O-CH, NH_2_-CH_2_ | 3.13(t), 3.81(t) |
| 33 | Choline | N(CH_3_)_3_, N-CH_2_ | 3.20(s), 3.51(m) |
| 34 | Phosphocholine | N(CH_3_)_3_, N-CH_2_, CH_2_ | 3.22(s), 3.59(t) , 4.15(m) |
| 35 | Taurine | NH_2_-CH_2_, SO_3_-CH_2_ | 3.26(t), 3.43(t) |
| 36 | TMAO | CH_3_ | 3.25(s) |
| 37 | Methanol | CH_3_ | 3.36(s) |
| 38 | Glycine | CH_2_ | 3.57(s) |
| 39 | N-phenylacetylglycine | N-CH_2_, CH_2_, CH=CH | 3.68(s), 3.76(d), 7.35(m), 7.38(m), 7.41(s) |
| 40 | Guanidoacetate | CH_2_ | 3.78(s) |
| 41 | Cysteine | βCH_2_, αCH | 3.06(m), 3.97(dd) |
| 42 | Creatinine | N-CH_3_, CH_2_ | 3.05(s), 4.07(s) |
| 43 | Allantoin | NH-CH-NH | 5.35(s) |
| 44 | Maleate | CH=CH | 6.02(s) |
| 45 | Deoxycytidine | CH=CH-N, CH_2_, CH | 2.38(m), 3.79(m), 4.03(dt), 4.45(dt), 5.88(d), 6.27(t), 7.83(d) |
| 46 | Fumarate | CH=CH | 6.53(s) |
| 47 | Trans-aconitate | αCH, γCH_2_ | 3.44(s), 6.60(s) |
| 48 | Gallate | CH=CH | 7.06(s) |
| 49 | 1-Methylhistidine | N=CH, CH=CH, CH_2_, CH-NH_2_ | 3.07(dd), 3.16(dd), 3.68(s), 3.96(dd), 7.01(s), 7.67(s) |
| 50 | 3-Indoxylsulfate | N=CH, CH=CH | 7.22(dd), 7.28(dd), 7.36(s), 7.51(d), 7.71(d) |
| 51 | 3-Phenyllactate | αCH, βCH_2_, CH=CH | 2.87(dd), 3.09(dd), 4.26(dd), 7.33(m) |
| 52 | Phenylalanine | CH=CH | 3.13(m), 3.28(m), 4.00(m), 7.32(d), 7.36(m), 7.43(m) |
| 53 | Hippurate | CH=CH, CH_2_ | 3.97(d), 7.55(t), 7.64(t), 7.84(d) |
| 54 | Benzoate | CH=CH | 7.49(dd), 7.56(t), 7.88(d) |
| 55 | Pyridoxine | CH_3_, CH_2_, CH=CH | 2.45(s), 4.73(s), 7.65(s) |
| 56 | Formate | HO-CH=O | 8.47(s) |
| 57 | Trigonelline | N-CH_3_, N=CH, CH=CH | 4.44(s), 8.07(m), 8.83(m), 9.11(s) |
| 58 | 1-Methylnicotinamide | N-CH_3_, N=CH, CH=CH | 4.44(s), 8.16(d), 8.88(d), 8.95(d), 9.26(s) |

Multiplicity: singlet (s), doublet (d), triplet (t), doublet of doublets (dd), quartets (q), multiplet (m).

Table S2 Levels of integrated areas of metabolites in cholestatic injury rats induced by thioacetamide (TAA) relative to normal control rats (NC) as depicted by z-scores (z-scores are colored for easy interpretation).


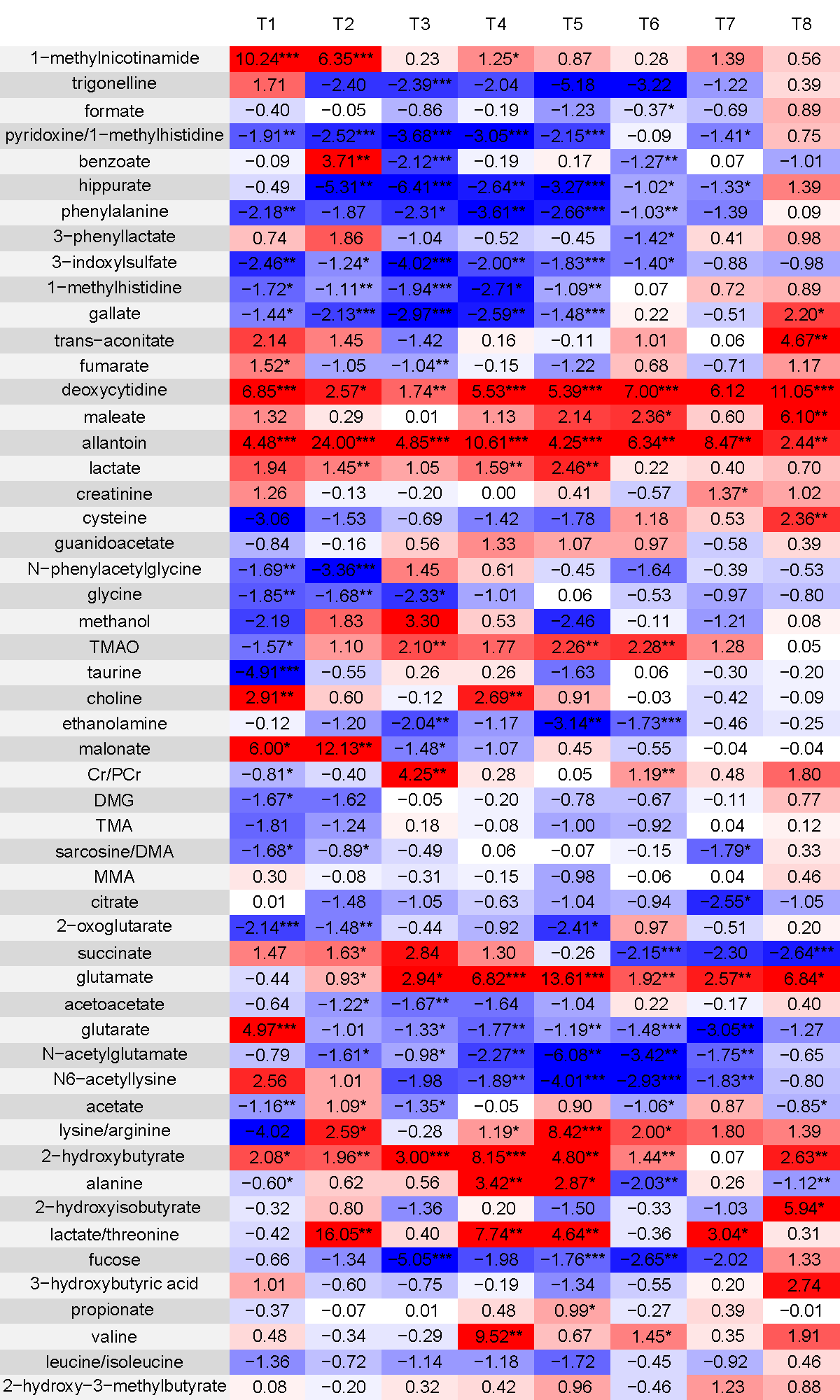


z-score = (mean_TAA_-mean_NC_)/SD_NC_

*p<0.05, **p<0.01, ***p<0.001: compare to NC group

Color code **
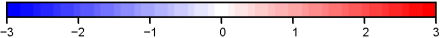
**

Table S3 Levels of integrated areas of metabolites in cholestatic injury rats induced by thioacetamide treated with HLJDD (THC) relative to normal control rats (NC) as depicted by z-scores.

**
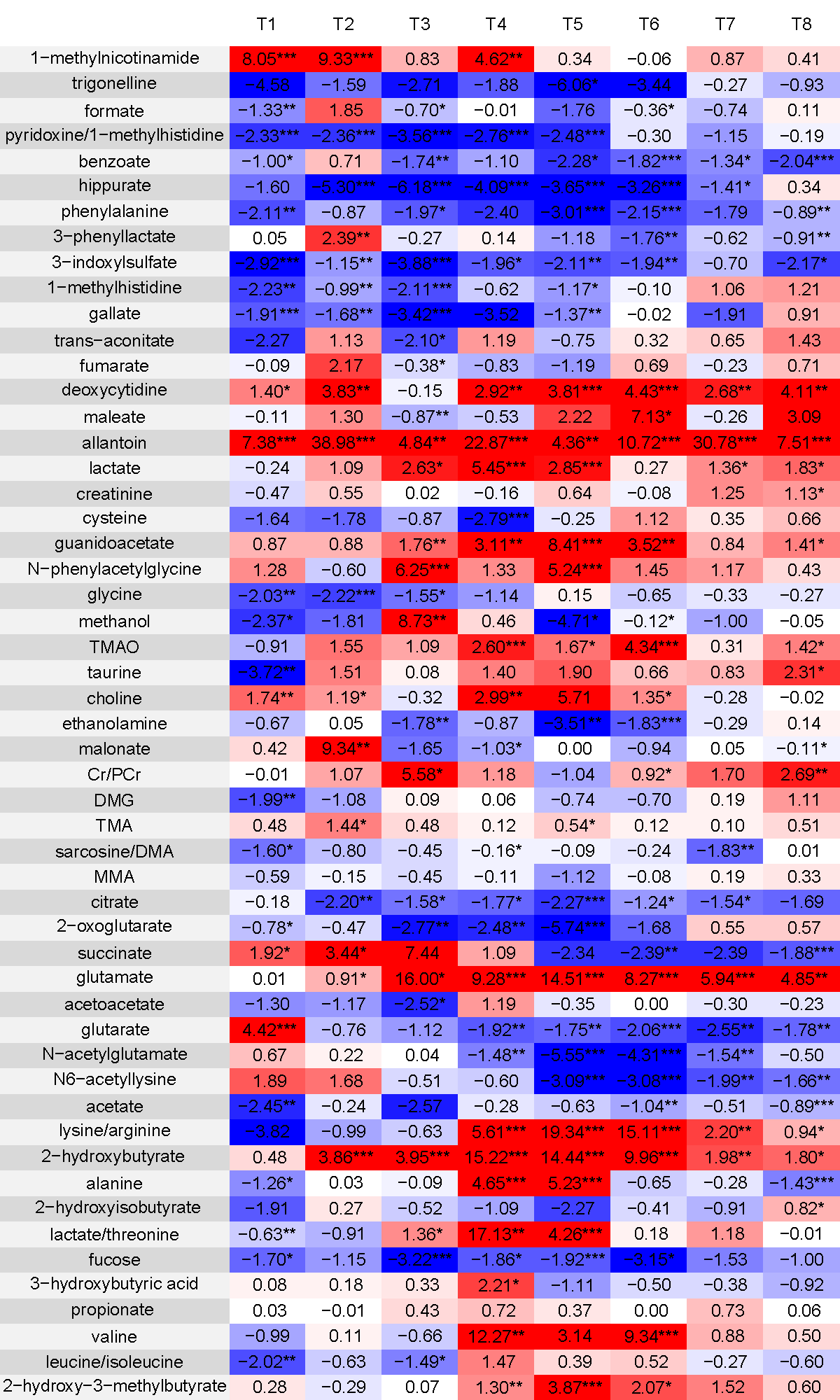
**

z-score = (mean_THC_-mean_NC_)/SD_NC_

*p<0.05, **p<0.01, ***p<0.001: compare to NC group

Color code **
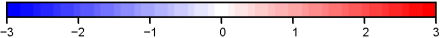
**

Table S4 Levels of integrated areas of metabolites in cholestatic injury rats induced by thioacetamide treated with HLJDD (THC) relative to HLJDD administrated healthy rats (HLD) as depicted by z-scores.

**
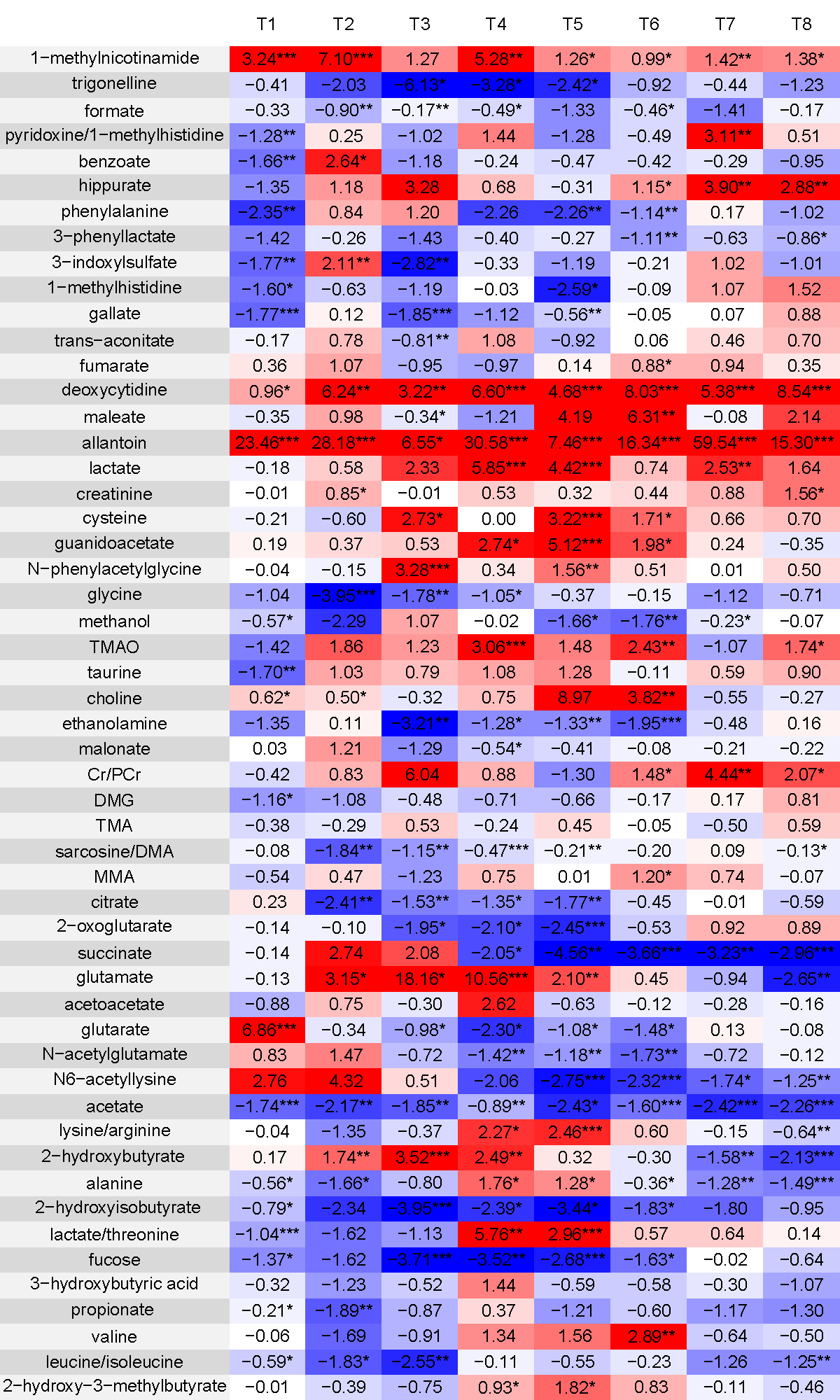
**

z-score = (mean_THC_-mean_HLD_)/SD_HLD_

*p<0.05, **p<0.01, ***p<0.001: compare to HLD group

Color code **
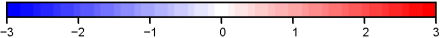
**

Table S5 Levels of integrated areas of metabolites in cholestatic injury rats induced by bile duct ligation (BDL) relative to normal control rats (NC) as depicted by z-scores.


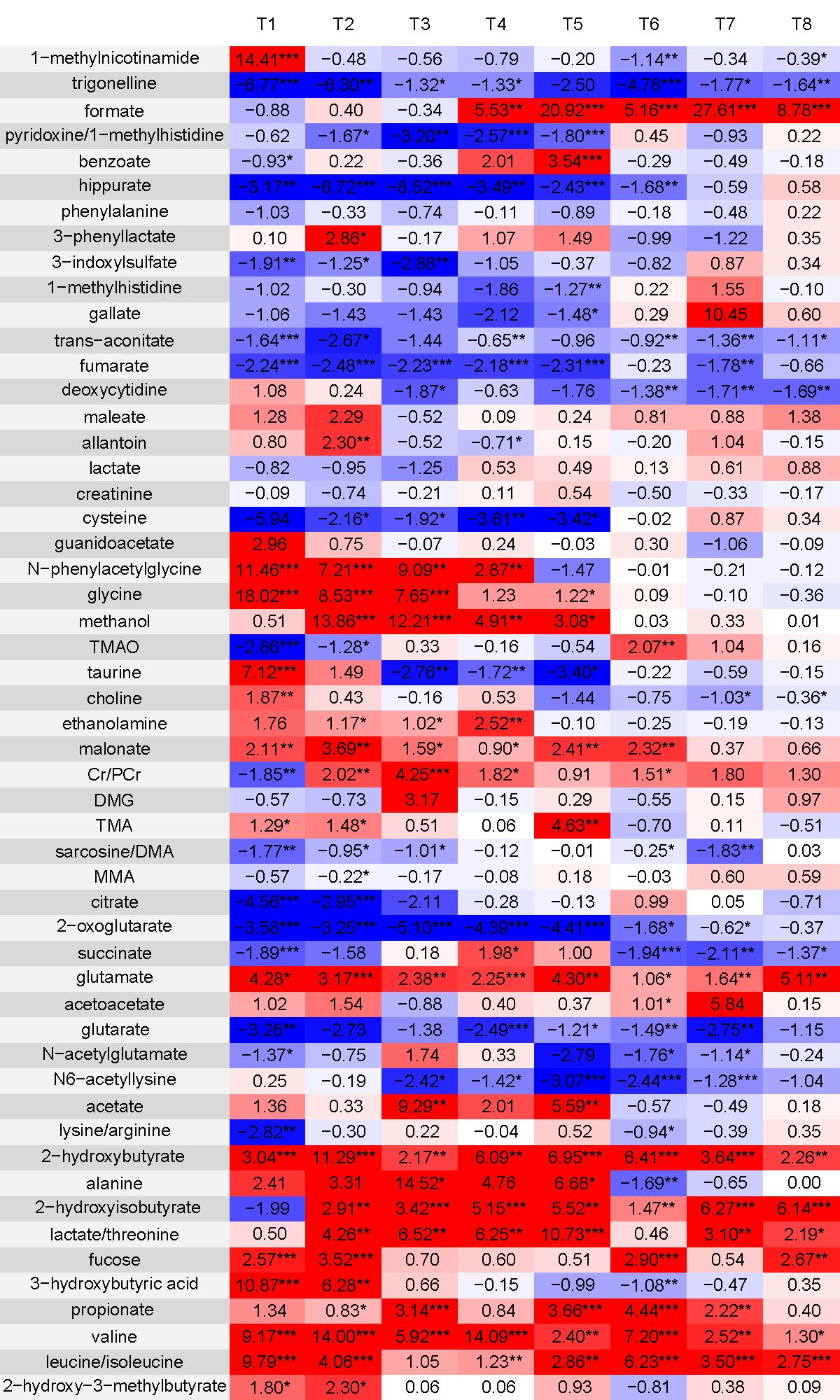


z-score = (mean_BDL_-mean_NC_)/SD_NC_

*p<0.05, **p<0.01, ***p<0.001: compare to NC group

Color code **
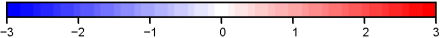
**

Table S6 Levels of integrated areas of metabolites in cholestatic injury rats induced by bile duct ligation treated with HLJDD (BHD) relative to normal control rats (NC) as depicted by z-scores.


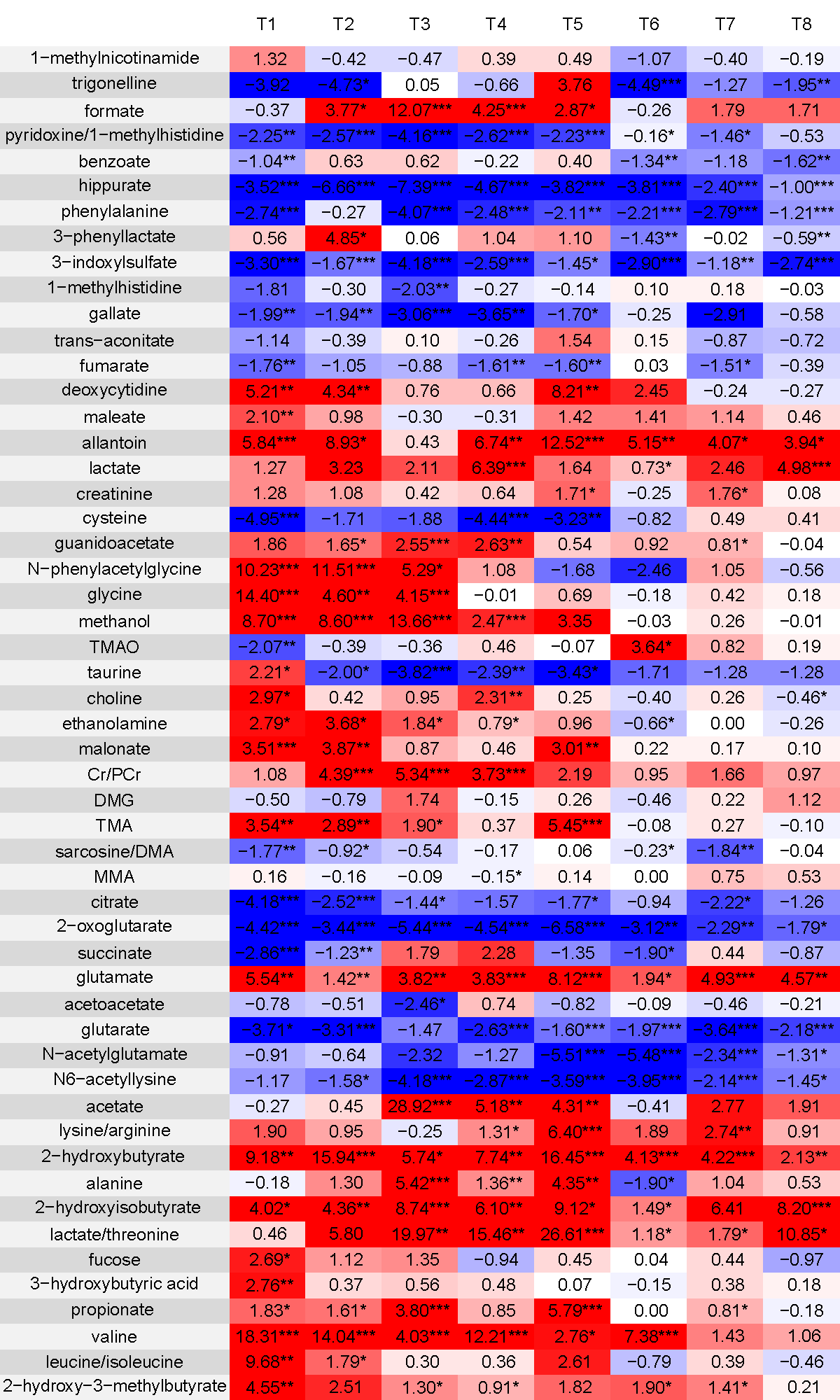


z-score = (mean_BHD_-mean_NC_)/SD_NC_

*p<0.05, **p<0.01, ***p<0.001: compare to NC group

Color code **
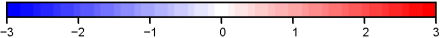
**

Table S7 Levels of integrated areas of metabolites in cholestatic injury rats induced by bile duct ligation treated with HLJDD (BHD) relative to HLJDD administrated healthy rats (HLD) as depicted by z-scores.


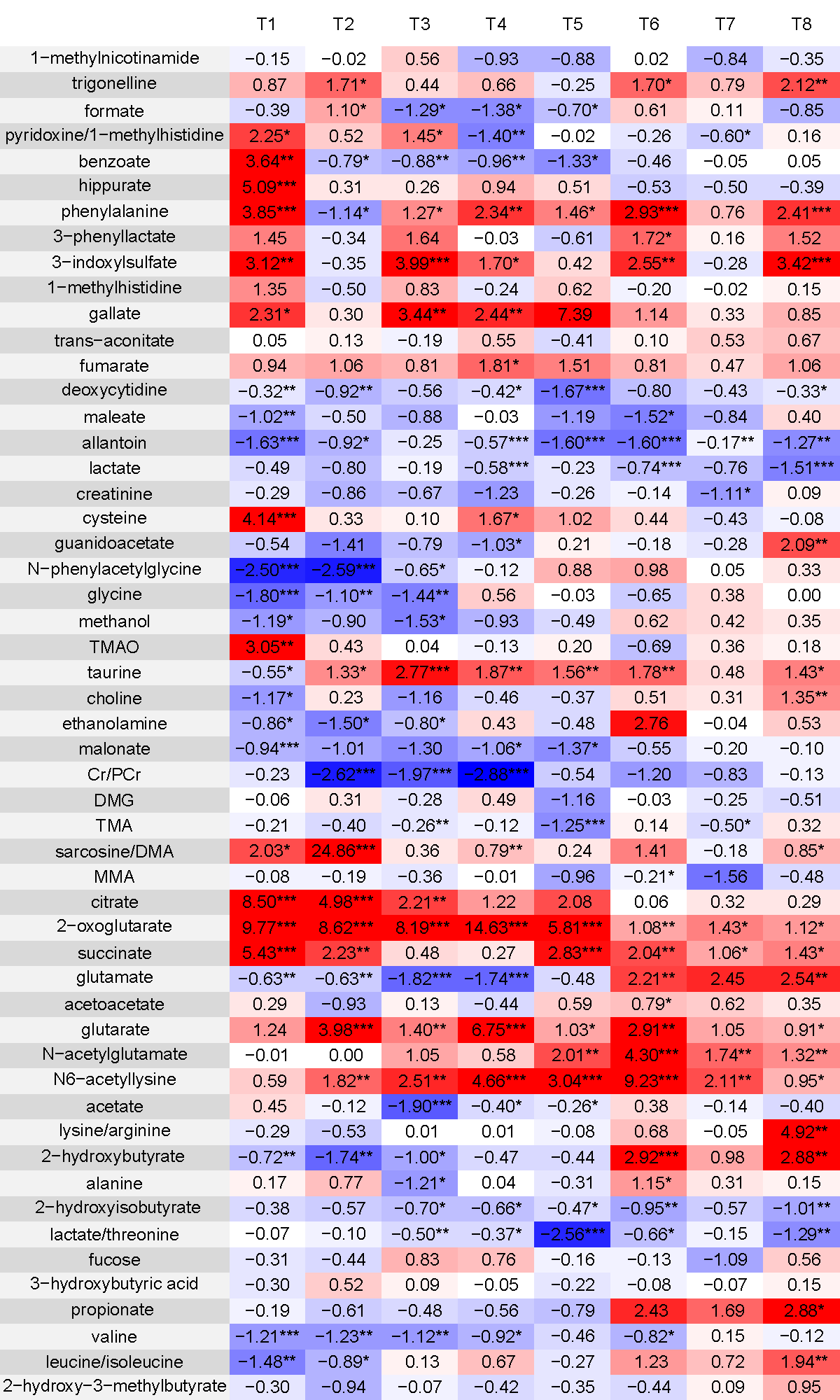


z-score = (mean_BHD_-mean_HLD_)/SD_HLD_

*p<0.05, **p<0.01, ***p<0.001: compare to HLD group

Color code **
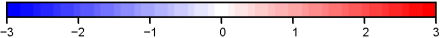
**
